# A complementary chemical probe approach towards customized studies of G-quadruplex DNA structures in live cells†

**DOI:** 10.1039/d1sc05816a

**Published:** 2022-02-01

**Authors:** Bagineni Prasad, Mara Doimo, Måns Andréasson, Valentin L’Hôte, Erik Chorell, Sjoerd Wanrooij

**Affiliations:** Department of Chemistry, Umeå University 90187 Umeå Sweden erik.chorell@umu.se; Department of Medical Biochemistry and Biophysics, Umeå University Umeå 90736 Sweden sjoerd.wanrooij@umu.se

## Abstract

G-quadruplex (G4) DNA structures are implicated in central biological processes and are considered promising therapeutic targets because of their links to human diseases such as cancer. However, functional details of how, when, and why G4 DNA structures form *in vivo* are largely missing leaving a knowledge gap that requires tailored chemical biology studies in relevant live-cell model systems. Towards this end, we developed a synthetic platform to generate complementary chemical probes centered around one of the most effective and selective G4 stabilizing compounds, Phen-DC3. We used a structure-based design and substantial synthetic devlopments to equip Phen-DC3 with an amine in a position that does not interfere with G4 interactions. We next used this reactive handle to conjugate a BODIPY fluorophore to Phen-DC3. This generated a fluorescent derivative with retained G4 selectivity, G4 stabilization, and cellular effect that revealed the localization and function of Phen-DC3 in human cells. To increase cellular uptake, a second chemical probe with a conjugated cell-penetrating peptide was prepared using the same amine-substituted Phen-DC3 derivative. The cell-penetrating peptide conjugation, while retaining G4 selectivity and stabilization, increased nuclear localization and cellular effects, showcasing the potential of this method to modulate and direct cellular uptake *e.g.* as delivery vehicles. The applied approach to generate multiple tailored biochemical tools based on the same core structure can thus be used to advance the studies of G4 biology to uncover molecular details and therapeutic approaches.

## Abstract 2

The development of an approach to generate multiple tailored biochemical tools based on the same core structure, Phen-DC3, to advance studies of G4 biology to uncover molecular details and therapeutic approaches.

## Graphical abstract

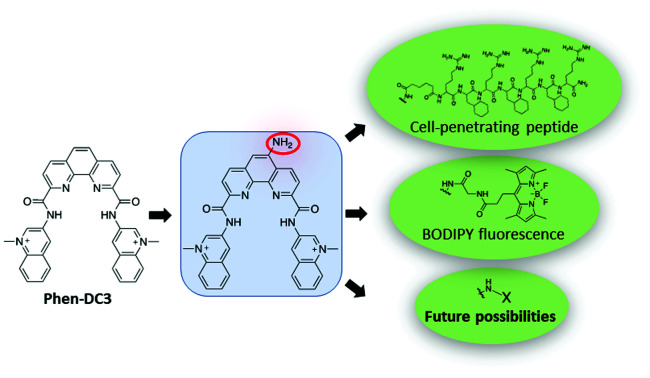

## Introduction

G-quadruplex (G4) DNA structures are four-stranded DNA structures that lately have gained significant scientific interest because of their links to key cellular events. The first report related to G4 DNA structures dates back to 1910 when Ivar Christian Bang mentioned that guanylic acid can form a gel at higher concentrations.^1^ In 1962, Martin Gellert *et al.* followed up on this work and published the first structure of a G-tetrad.^2^ At that time, the G4 DNA structure was believed to be an *in vitro* artefact and research related to G4 DNA was for long not prioritized. However, a growing body of evidence has lately shown that G4 DNA structures are highly abundant, evolutionary conserved, and seem to play important roles in the processing of our genetic information.^3,4^ The core of a G4 structure consists of layers of guanines that stack on each other. Each guanine layer, called a G-quartet or G-tetrad, consists of four guanines that bind to each other by Hoogsteen hydrogen bonding.^2,5^ The G4 structures are further stabilized by monovalent cations,^6–8^ and can altogether form a structure that is more stable than dsDNA.^9^ However, the stability of a G4 structure is highly dependent on the direction of the four G-strands that form the central guanine core and the composition, length, and position of the nucleotide sequences connecting the G-strands, called loops. Although the guanine core structure is similar between G4 structures, the loops can be highly variable and thus confer large structural diversity between G4 structures.^10–12^ Furthermore, G4 structures can be formed intramolecular (by one DNA strand) or intermolecular (by two or four individual strands) and present a diversity of topologies as defined by the parallel or antiparallel strand orientation.^13^ Recent genomic approaches identified at least 700 000 G4 structures in the genome of human cells.^14^ Their location is distributed throughout the human genome, but they are enriched at promoters and specifically in the promoters of many oncogenes such as c-MYC, BCL2, VEGF, and c-KIT.^15,16^ G4 structures are thus considered promising drug targets and are investigated in different therapeutic strategies to treat various cancers.^17–19^ The abundance of G4 structures and their enrichment at certain genomic locations are strong indications of their cellular importance. Studies have identified potential regulatory roles of G4s in replication, transcription, splicing, translation, and recombination.^4,14^ In addition to this, G4 structures also impact the mitochondrial DNA gene expression. Putative G4-forming sequences are found all around the 16kb-long circular DNA molecule that shapes the human mitochondrial genome,^20,21^ moreover G4 structures have a functional role during mitochondrial transcription.^22,23^ Nonetheless, the precise influence of G4 structures on mitochondrial DNA maintenance is still largely unknown. Taken together, there are vast amounts of G4 structures that possess broad structural diversity, and their presence is linked to key biological processes. However, there are still considerable gaps in the knowledge of G4 biology and new techniques and strategies to study G4 biology are of immense interest.

Chemical biology is a research field at the interface of chemistry and biology that generally involves manipulation and studies of biology using small molecules. One such example is the generation and validation of new small-molecule-based tool compounds. These serve as excellent tools to modulate biological systems and possess unique characteristics that have proven highly valuable to *e.g.* study G4 biology. Indeed, one way to study G4 DNA structures is to develop small molecules that bind and stabilize G4 structures. Phen-DC3 is one of the most frequently used and strongest G4 stabilizing compounds.^24^ There are many exquisite studies that focus on the development of a particular chemical probe for studies of G4 structures.^25–28^ In here, we develop and explore a method based on the use of, not a single, but several complementary chemical probes to enable tailored studies of G4 structures. To achieve this, we develop a synthetic platform based on Phen-DC3 that allows for customized functionalization with different complementary handles for *e.g.* pull-downs, fluorescence studies, target delivery peptides, etcetera. The utility of this synthetic platform is exemplified by the introduction of a 4,4-difluoro-4-bora-3*a*,4*a*-diaza-*s*-indacene (BODIPY) fluorophore that revealed the basis for the limited cellular effect of Phen-DC3 despite its strong G4 binding and stabilization. Based on this, the diversity of the approach is further exemplified by the conjugation of a cell-penetrating peptide that effectively opens the possibility for cellular studies. Importantly, even though the conjugated functionalities are large and charged, their presence does not alter the potent and selective G4 stabilization of Phen-DC3. These studies thus show the power of the presented strategy to introduce complementary chemical probes on the same starting compound to advance chemical biology studies aimed at G4 biology.

## Results and discussions

**Fig. 1:**
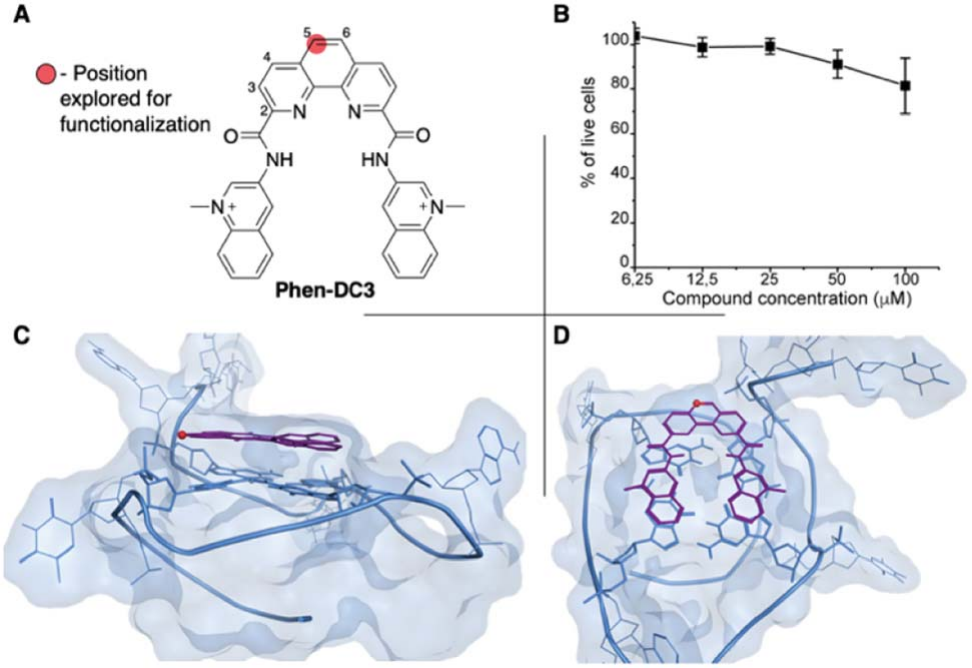
(A) Chemical structure of Phen-DC3. (B) Resazurin-based cell viability assay on HeLa cells treated for 48 h with Phen-DC3 at the indicated concentrations. Data represent mean ± standard deviation of three independent experiments. (C and D) Crystal structure (2MGN) of Phen-DC3 (purple) and Pu24T (blue) viewed from the side (C) and top (D). The carbon labelled with a red sphere in (A), (C), and (D) represents the position on the phenanthroline core suitable for functionalization.

Phen-DC3 is one of the strongest G4 stabilizers reported (Fig. 1A). *In vitro*, Phen-DC3 can stabilize with high affinity and specificity G4 structures of different topologies.^24,29^ However, in HeLa cells, the effect of Phen-DC3 on cell viability is surprisingly mediocre, with only 20% cell death after 100 μM treatment for 48 h (Fig. 1B).

**Scheme 1:**
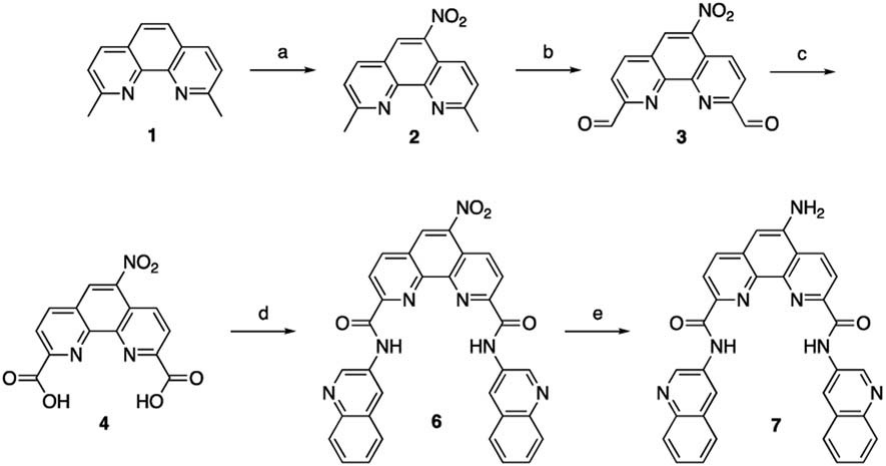
Synthesis of amino substituted Phen-DC3. Conditions: (a) HNO_3_, H_2_SO_4_, 120 °C, 1 h, 44%; (b) SeO_2_, 1,4-dioxane:H_2_O (0.4%), reflux, 3 h; (c) Conc. HNO_3_, reflux, 6 h, (2 steps 56%); (d) 3-aminoquinoline 5, HATU, DIPEA, DMF, rt, 12 h, 87%; (e) Pd/C, H_2_, 80 °C, DMF, 3 h, 72%.

To address the limited effect of this commercially available, well-characterized, and recognized G4 stabilizing compound on cell viability, we set out to develop a synthetic methodology, which has not been described to date. Such a strategy would allow for the functionalization of Phen-DC3 with various versatile substituents for customized cellular studies without affecting the integrity of Phen-DC3 and its potent and selective G4 stabilization. For this purpose, we investigated reported structural data on how Phen-DC3 interacts with G4 DNA structures, *e.g.* the structure of Phen-DC3 bound to the Pu24T *c-MYC* G4 DNA structure.^30^ This revealed positions 5 and 6 on the central phenanthroline scaffold as excellent connection points for further functionalization of Phen-DC3 without affecting the G4 binding properties (Fig. 1C and D). Hence, the preparation of a Phen-DC3 derivative with a primary amine in this position would open up for further functionalization. Thus, we developed synthetic methods to this key derivative starting by selective nitration of Neocuproine 1 using a combination of fuming sulfuric acid and nitric acid at 120 °C to generate nitro derivative 2 (Scheme 1). Compound 2 was next converted to dicarbaldehyde 3 by selenium mediated oxidation, which was further oxidized to the dicarboxylic acid derivative 4 in 56% overall yield.^31^ A coupling reaction between acid 4 and 5 (3-aminoquinoline) generated compound 6 in 87% yield using 1-[bis(dimethylamino)methylene]-1*H*-1,2,3-triazolo[4,5-*b*]pyridinium 3-oxide hexafluorophosphate (HATU) and *N*,*N*-diisopropylethylamine (DIPEA) at room temperature. Finally, a reduction of the nitro functionality in compound 6 using a Pd/C mediated hydrogenation generated the desired primary amine 7 in 72% yield.

**Scheme 2:**
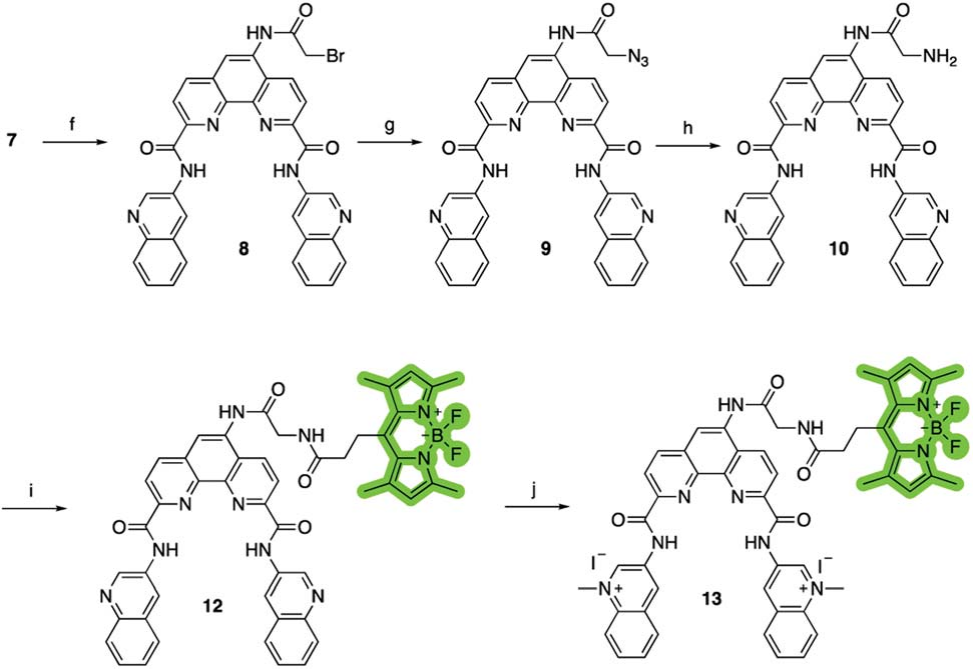
Synthesis of Phen-DC3 linker with BODIPY. Conditions: (f) bromoacetyl chloride, Et_3_N, rt, 4 h, 80%; (g) NaN_3_, DMF, rt, 70%; (h) PPh_3_, THF, H_2_O, 90 °C, 8 h, 72%; (i) 3-(5,5-difluoro-1,3,7,9-tetramethyl-5*H*-4λ4,5λ4 di-pyrrolo[1,2-*c*:2′,1′-*f*][1,3,2]diaza-borinin-10-yl)propanoic acid 11, HATU, DIPEA, DMF, rt, 1 h, 65%; (j) MeI, DMF, 40 °C, 24 h, 70%.

To investigate the cellular localization of Phen-DC3 in live cells, we used the developed synthetic methodology to link a BODIPY fluorophore to Phen-DC3 (Scheme 2). The BODIPY fluorophore was chosen because of its high quantum yield of fluorescence, absorption/emission wavelengths, photostability, and relatively small size. Hence, bromoacetyl chloride was reacted with amine 7 in the presence of DIPEA to give bromoderivative 8, which upon treatment with sodium azide provided compound 9. Staudinger reduction conditions effectively reduced the azido derivative 9 to amine derivative 10, and a subsequent HATU mediated coupling reaction with BODIPY acid^32^11 successfully provided the BODIPY linked Phen-DC3 13 after methylation using methyl iodide.

A Taq polymerase stop assay was next performed to investigate if the addition of the BODIPY fluorophore would affect the ability of Phen-DC3 to selectively stabilize G4 DNA. This assay measures how efficiently the Taq polymerase can synthesize new DNA from a template strand with a single nucleotide resolution readout by extending a fluorescent 5′ end-labelled DNA primer. A G4 DNA structure is an obstacle for the DNA polymerase, and if a template strand with a G4 DNA structure is used, the Taq polymerase will halt one or two nucleotides before the first G-tetrad in the G4 structure.

**Fig. 2:**
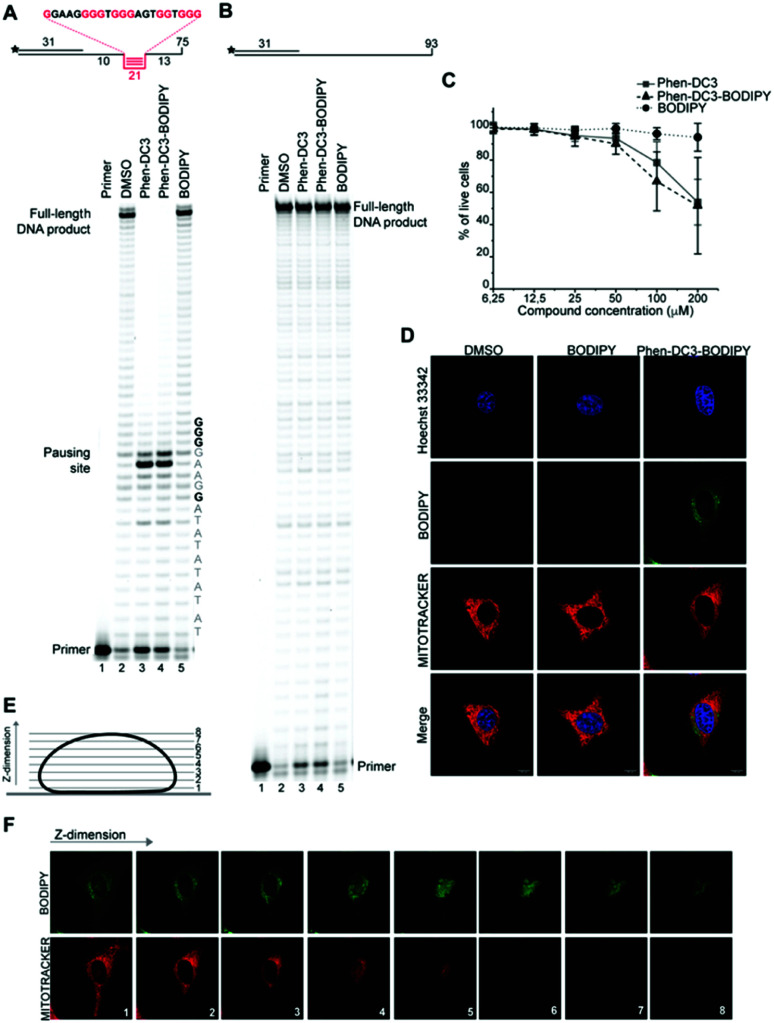
Phen-DC3-BODIPY has the same effect than Phen-DC3 *in vitro* and in HeLa cells and does not enter the nucleus. (A) Taq-polymerase STOP assay on a template with a G4 structures (as depicted above the gel) in the presence of 1 μM of the indicated compounds. The nucleotides of the template upstream and around the pausing site are indicated. Guanines involved in the formation of the G4 are indicated in bold. (B) Taq-polymerase STOP assay on a non-G4 DNA template in the presence of 1 μM of the indicated compounds. (C) Resazurin-based cell viability assay on HeLa cells treated for 48 h with Phen-DC3, Phen-DC3-BODIPY or BODIPY at the indicated concentrations in high glucose medium. Data represent mean ± standard deviation of three independent experiments. (D) Planar confocal images of HeLa cells treated for 12 h with 5 μM of the indicated compounds. Cells were trypsinized, washed and allowed to seed again in on new coverslip for approximately 6 h before imaging. Mitochondria were stained with Mitotracker red, nuclei were stained with Hoechst 33342. (E) Schematic representation of the Z-stacks acquisition at the confocal microscopy. (F) Single Z-stacks of HeLa cells treated with 5 μM Phen-DC3-BODIPY. Mitochondria were stained with Mitotracker red. The numbers indicate the single stacks as represented in (E).

However, the DNA polymerase can fairly easily resolve the G4 structure and synthesize the full-length run-off DNA product. The efficiency of G4 stabilizing compounds can be measured by their ability to increase the stalling of the DNA polymerase at the G4 structure on the template strand. Also, it provides nucleotide resolution of the DNA polymerase stalling. We performed these primer extension assays in the presence of DMSO (neg. control), Phen-DC3 alone, BODIPY alone, and Phen-DC3 linked to BODIPY using a G4 (Fig. 2A) and a non-G4 (Fig. 2B) containing DNA template. In the absence of a G4 stabilizer (DMSO) the DNA polymerase could extend the DNA primer beyond the G4 structure in the DNA template to generate a full-length run-off DNA product (Fig. 2A, lane2). As expected, the addition of Phen-DC3 led to a strong stabilization of the template G4, resulting in a clear DNA polymerase stalling before the G4 structure (Fig. 2A lane 3). Interestingly, the BODIPY linked Phen-DC3 retained its G4 stabilization ability (Fig. 2A, lane 4), whereas the BODIPY fluorophore alone did not impact the activity of the DNA polymerase (Fig. 2A lane 5).

Similar to Phen-DC3 alone, the BODIPY linked Phen-DC3 retained its G4 selectivity and displayed no effect in the DNA polymerase stop assay when a template strand without G4 structure was used in the reaction (Fig. 2B, lane 3 and 4). In addition, a fluorescence resonance energy transfer (FRET) assay was performed to further validate the results obtained from the Taq polymerase stop assay. In this assay, we measure the differences in the melting temperature (Δ*T*_m_) of fluorescently labelled G4 DNA in the presence and absence of BODIPY linked Phen-DC3 to investigate its ability to stabilize this G4 structure at different concentrations (1, 2, 5 and 8 μM). In accordance with the Taq-polymerase STOP assay, the FRET assay showed that BODIPY linked Phen-DC3 was able to efficiently stabilize G4 DNA and also displayed high selectivity towards G4 DNA over dsDNA (ESI Fig. 1A†). To investigate the selectivity of BODIPY linked Phen-DC3 between different G4 topologies, we measured its ability to stabilize different G4 structures using FRET. This showed a similar selectivity as Phen-DC3 alone although a discrimination against cKIT G4 DNA could be observed (ESI Fig. 1B†).^33^ We also used circular dichroism (CD) spectroscopy and ^1^H-NMR titrations to investigate if the compound affects the G4 conformation, which showed that BODIPY linked Phen-DC3 does not induce any major conformational changes of the G4 DNA structure (ESI Fig. 2†).

To further verify that the BODIPY linking strategy does not affect the cellular properties of Phen-DC3, we performed cell viability studies, which revealed near to identical effects of Phen-DC3-BODIPY compared to treatment with Phen-DC3 alone (Fig. 2C). BODIPY alone had minimal effects on cell viability (Fig. 2C). We conclude that introducing the BODIPY fluorophore to Phen-DC3 using our synthetic platform has generated a fluorescent Phen-DC3 derivative that has retained *in vitro* and cell viability effects of Phen-DC3. This allowed us to use fluorescence live-cell imaging experiments to study the localization of this compound. Cells were treated with 5 μM of BODIPY linked Phen-DC3 for 12 h, washed, and allowed to adhere for a period of 6 h before live-cell studies (Fig. 2D).

**Fig. 3:**
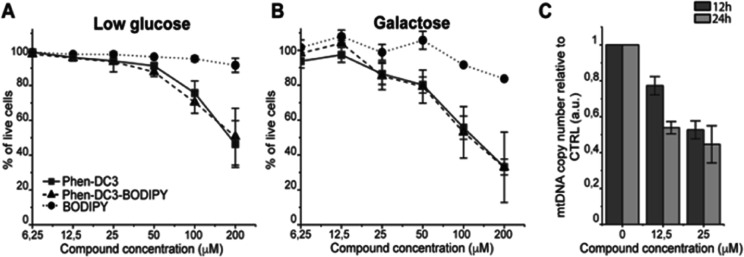
Phen-DC3 affects mtDNA copy number in HeLa cells. Resazurin-based cell viability assay on HeLa cells treated for 48 h with Phen-DC3, Phen-DC3-BODIPY or BODIPY at the indicated concentrations in low glucose medium (A) or in galactose medium (B). Data represent mean ± standard deviation of three independent experiments. (C) mtDNA copy number of HeLa cells treated for 12 h or 24 h with Phen-DC3 at the indicated concentrations. MtDNA copy number was determined by quantitative PCR from total DNA. The relative mtDNA copy number was expressed as the ratio between mtDNA ATP6 region and nuclear DNA r18S region. Data represent mean ± abs error of two independent experiments.

This revealed that Phen-DC3-BODIPY does enter the cells but, strikingly, that there is no presence of BODIPY linked Phen-DC3 in the nucleus (no-colocalization with Hoechst 33342 (nuclear) staining) (Fig. 2D). This fact could explain the minimal effect of Phen-DC3 on cell viability, despite it being a highly selective and efficient G4 stabilizer that should have detrimental effects when localized to the nucleus (Fig. 1B and 2C). Interestingly, some overlap with the mitochondrial staining (Mitotracker) is observable, suggesting that Phen-DC3 might enter mitochondria (Fig. 2E and F). Based on this observation, we hypothesized that a mitochondrial localization of Phen-DC3 should lead cells to be more sensitive when maintained in growth conditions that depend on mitochondrial function. Indeed, HeLa cells were more sensitive to Phen-DC3 treatment when dependent on mitochondrial energy production (galactose or low glucose medium) compared to highly glycolytic cells (grown in high glucose-medium) (compare Fig. 1B to Fig. 3A and B). The Phen-DC3-BODIPY derivative showed identical results compared to Phen-DC3 alone, which again underlines the value of the developed approach to link chemical probes to Phen-DC3 without affecting its interactions with G4 DNA.

Because Phen-DC3 is highly selective for G4 DNA binding and stabilization *in vitro*, we hypothesize that its target in the mitochondria is the mitochondrial DNA (mtDNA) and its potential G4 structures. Formation and stabilization of G4 structures can lead to stalling of the mtDNA replication machinery and partial loss of the total mtDNA pool. To investigate this, we looked at the effect of Phen-DC3 treatment on the mtDNA copy number in HeLa cells. We detected a clear Phen-DC3 concentration-dependent decrease in the mtDNA copy number after 12 h treatment (Fig. 3C). Extended Phen-DC3 treatment (24 hours) resulted in a further decreased mtDNA copy number (Fig. 3C). We conclude that Phen-DC3 can enter the mitochondria and bind to G-quadruplex structures on the mtDNA to impede mtDNA replication and reduce the mtDNA copy number. Taken together, our data suggest that Phen-DC3 has limited cellular uptake and is unable to enter the nucleus in live cells. The small amount of Phen-DC3 that is taken-up remains cytosolic and exerts its effect on the mitochondrial function by affecting the mtDNA maintenance process.

**Scheme 3:**
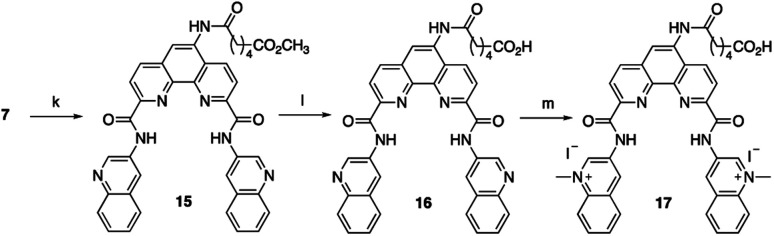
Synthesis of Phen-DC3 linker with acid. Conditions: (k) methyl 6-chloro-6-oxohexanoate 14, TBD, DCM, rt, 3 h, 82%; (l) 1 M LiOH, DMF, 3 h, 76%; (m) MeI, DMF, 40 °C, 18 h, 92%.

To increase the ability of Phen-DC3 to enter human cells and thus greatly expand its value as a research tool, we again turned to our developed synthetic methodology with the aim to conjugate a cell-penetrating peptide to Phen-DC3, Phen-DC3-PP, to increase cellular uptake of the compound. The central amine 7 was thus coupled with methyl adipoyl chloride 14 in the presence of 1,5,7-triazabicyclo[4.4.0]dec-5-ene (TBD) base to give methyl-6-oxohexanoate compound 15 in 82% yield. Ester hydrolysis to hexanoic acid derivative 16 was performed with lithium hydroxide in DMF in 76% yield followed by methylation using methyl iodide in DMF at 40 °C to give the target compound 17 in 92% yield (Scheme 3).

**Scheme 4:**
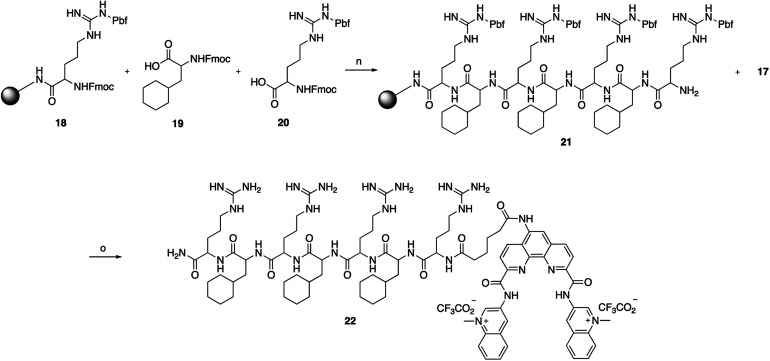
Synthesis of Phen-DC3 linker with PP. Conditions: (n) (i) HBTU, HOBT, DIPEA, DMF, rt; (ii) piperidine; (o) (i) 17, HATU, DIPEA, DMF, rt, 12 h, 87%; (ii) TFA : TIPS : H_2_O (15 : 1 : 1), rt, 2h.

The key Phen-DC3 derivative 17 was next linked with the PP as outlined in Scheme 4. The couplings were performed on rink amide resin with 2,2,4,6,7-pentamethyldihydrobenzofuran-5-sulfonyl (Pbf) protected arginine (18) using amino acids cyclohexyl alanine 19 and arginine 20. HBTU and HOBT were used as coupling agents and DIPEA as the base in DMF on a solid phase synthesizer. Acid derivative 17 was subsequently coupled to the N-terminus of the resin-bound PP^34^ (rF_*x*_rF_*x*_rF_*x*_r; where r = d-arginine and F_*x*_ = l-cyclohexylalanine) with HATU as the coupling reagent and DIPEA as the base in DMF. The target Phen-DC3 linked to PP (22) was cleaved from the resin with a TFA : TIPS : H_2_O (15 : 1 : 1) mixture and then precipitated by the addition of cold diethyl ether (Scheme 4).

**Fig. 4:**
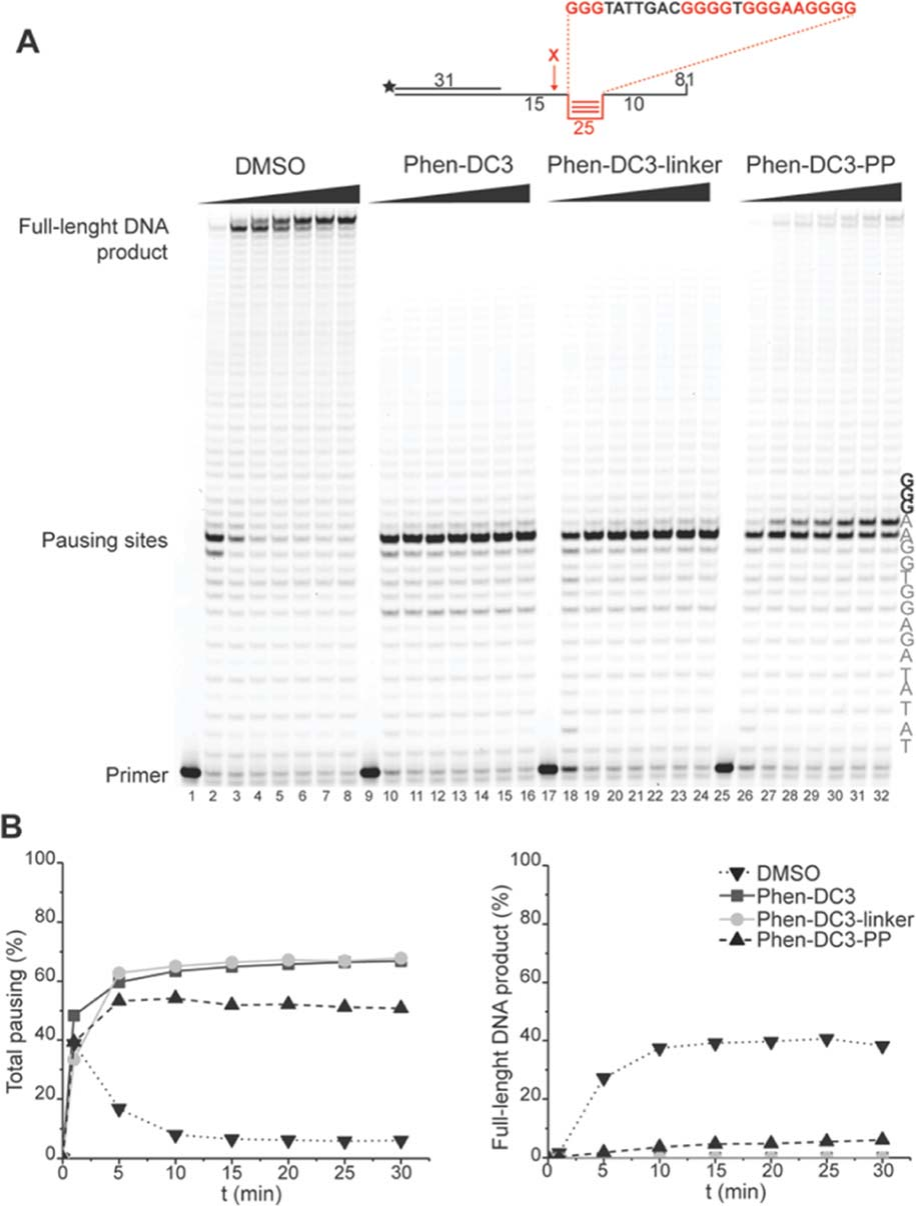
Fusion of the penetrating peptide does not affect Phen-DC3 ability to stabilize G4. (A) Taq-polymerase STOP assay on a template with a G4 structure in the presence of 250 nM of the indicated compounds. For each compound, the reaction was blocked at increasing time points. The nucleotides of the template upstream and around the pausing site are indicated. Guanines involved in the formation of the G4 are indicated in bold. (B) Quantification of pausing sites (left) and full-length product (right). Values are represented as % of the total line signal.

To assess if the charged PP addition would affect the ability of Phen-DC3 to selectively stabilize G4 DNA, we performed the FRET melting assay. This shows that Phen-DC3-PP (22) is still able to stabilize G4 DNA with a similar selectivity pattern as BODIPY linked Phen-DC3 (13) and that Phen-DC3-PP (22) does not affect dsDNA (ESI Fig. 4A and B†). However, the Phen-DC3-PP induced thermal stability of G4 DNA is decreased compared to BODIPY linked Phen-DC3 (13) (ESI Fig. 1A†). To further investigate this, we thus performed the Taq DNA polymerase stop assay which measures G4 stabilization at physiological temperatures. In this assay, Phen-DC3-PP (22) retains the ability to stabilize G4 DNA and blocks the DNA polymerase just upstream of the first G-tetrad on the G4 containing DNA template (Fig. 4A lanes 26-32). Compared to Phen-DC3 alone and Phen-DC3 with only the linker (ESI Scheme S1†), the attachment of the large and charged PP does have a surprisingly low effect on G4 DNA stabilization, and only a slight difference in the G4 stabilization is observed (Fig. 4A compare lanes 10–24 with lanes 26–32 and ESI Fig. 3A†). Although in the presence of Phen-DC3, some DNA products were detected that had extended DNA beyond the G4, this full-length run-off DNA product signal was strongly reduced compared to the negative control DMSO (6% *vs.* 38% after 30′) (Fig. 4B). Importantly, Phen-DC3-PP also retains its selectivity towards G4 DNA in this assay and does not affect DNA polymerase extension when a non-G4 DNA template is used (ESI Fig. 3B and C†). Furthermore, CD spectroscopy and ^1^H-NMR titrations showed that Phen-DC3-PP does not induce any major changes in the G4 topology (ESI Fig. 5†).

**Fig. 5:**
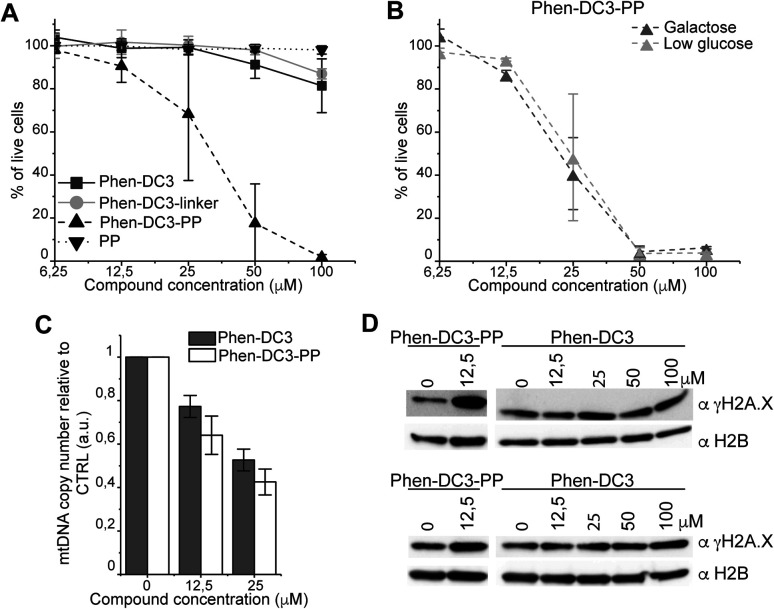
Addition of the penetrating peptide cause DNA damage in HeLa cells. Cell viability assay on HeLa cells treated for 48 h with the compounds at the indicated concentrations in high glucose medium (A) or in low glucose or galactose media (B). Data represent mean ± standard deviation of three independent experiments. (C) mtDNA copy number of HeLa cells treated for 12 h with Phen-DC3 or Phen-DC3-PP at the indicated concentrations. MtDNA copy number was determined by quantitative PCR from total DNA. The relative mtDNA copy number was expressed as the ratio between mtDNA ATP6 region and nuclear DNA r18S region. Data represent mean ± abs error of two independent experiments. (D) Immunoblot analysis of chromatin-bound protein fractions extracted from HeLa cells treated for 12 h with Phen-DC3 or Phen-DC3-PP at the indicated concentrations. Two independent biological replicates are shown.

To investigate how the ability to stabilize G4 DNA *in vitro* translates into their effect on cells, we next performed cell viability assays. Indeed, Phen-DC3-PP has a significantly increased effect on cell viability compared to Phen-DC3 alone, which is in line with an increased cellular uptake of PP-linked Phen-DC3 (Fig. 5A).

Importantly, the observed effects on cell viability are linked to Phen-DC3 and not to the cell-penetrating peptide as the peptide alone did not have any effect on cell viability (Fig. 5A). The stronger cytotoxicity of PhenDC3-PP did not correlate with an increased effect on mitochondrial function as cells treated with Phen-DC3-PP behaved similarly to those treated with Phen-DC3 alone when grown in conditions in which mitochondrial energy production is essential (low glucose or galactose-medium) (Fig. 5B). Furthermore, the effect on mtDNA copy number was also comparable between Phen-DC3-PP and Phen-DC3 alone treated HeLa cells (Fig. 5C). The increased effect on cell viability by conjugation of a cell-penetrating peptide can therefore possibly be explained by the ability of Phen-DC3-PP to enter the cell nucleus and stabilize nuclear G4 DNA. To investigate this, we analyzed the effect of Phen-DC3 and Phen-DC3-PP on nuclear DNA damage. We measured the phosphorylation of the histone variant H2A.X (named γH2A.X), an early cellular response to the induction of DNA double-strand breaks in the nucleus using a specific γH2A.X antibody.^35^ Indeed, Phen-DC3-PP induces a robust DNA damage response in HeLa cells already at 12.5 μM (Fig. 5D). By contrast, the DNA damage response induced by unmodified Phen-DC3 is moderate even after treatment with 100 μM (Fig. 5D). Conjugation of a cell-penetrating peptide to Phen-DC3 thus increases the cell penetration and the cytotoxic effect of Phen-DC3. This strategy enables studies of Phen-DC3 and its effect on nuclear DNA in cells, which is of high relevance considering that Phen-DC3 is among the most efficient and frequently used G4 DNA stabilizing compounds.

## Conclusions

Here, we develop a concept based on complementary chemical probes to allow for tailored studies of G4 biology. The concept was exemplified on the well-characterized G4-stabilizer Phen-DC3 through the combined use of different probes added on the same G4 ligand. To achieve this, we used structural-based design to functionalize Phen-DC3 with a reactive handle for further conjugations without interfering with G4 interactions. This required substantial synthetic method developments that was motivated by the general interest in this G4 ligand and the value of transforming it into different chemical probes for tailored studies of G4 biology.

We first linked a fluorophore to Phen-DC3 generating a fluorescent Phen-DC3 derivative that retained the *in vitro* activity and cellular functions of the original compound and enabled live-cell fluorescence studies. This allowed us to unveil that the reason behind the surprisingly low effects of Phen-DC3 on cell viability is its inability to enter the nucleus. Instead, Phen-DC3 partially localizes in the mitochondria where it is affecting the mtDNA copy number. The developed synthetic methodology was then used to link a cell-penetrating peptide to Phen-DC3. The penetrating peptide conjugation still retained G4 selectivity and stabilization *in vitro* but strongly increased the cellular effects. This is in line with an increased cellular and nuclear localization and opens up a whole new avenue for cell studies of G4 structures, *e.g.* by tuning the cell-penetrating peptide for use as delivery vehicles to also target specific tissues or tumors. Taken together, this shows that the developed synthetic methodology is highly flexible and allows for the conjugation of both large and charged functionalities without affecting the ability to very selectively and strongly stabilize G4 DNA. The presented work thus disentangles important insights regarding the cellular localization and function of Phen-DC3 and describes an approach that allows the introduction and application of an array of probes for various customized chemical biology studies of G4 DNA both *in vitro* and in the cells to study G4 biology.

## Data availability

The data supporting this article have been uploaded as part of the ESI.†

## Author contributions

P. B. developed the synthetic methods, synthesized and characterized the compounds; M. D. performed the cell viability, imaging studies, immunoblot analysis. M. A. performed and analysed the CD studies, the FRET assay, and the NMR studies; V. L. H. performed the Taq polymerase STOP assay and the mtDNA copy number studies; E. C. and S. W. initiated and directed the project; E. C., S. W., and M. D., wrote the manuscript but all authors read and edited the manuscript.

## Conflicts of interest

There are no conflicts to declare.

## Supplementary Material

SC-013-D1SC05816A-s001
