# Supplementary material for: A complementary chemical probe approach towards customized studies of G-quadruplex DNA structures in live cells
Source: Chem Sci. 2022 Feb 1;13(8):2347–54. doi: 10.1039/d1sc05816a (PMC8864707; doi:10.1039/d1sc05816a)
Supplement: SC-013-D1SC05816A-s001 [file SC-013-D1SC05816A-s001.pdf]

Electronic Supplementary Material (ESI) for Chemical Science.
This journal is © The Royal Society of Chemistry 2022

Electronic Supplementary Information

A complementary chemical probe approach towards customized studies of G-quadruplex DNA structures in live cells

Bagineni Prasad[a, ‡], Mara Doimo [b, ‡], Måns Andréasson[a], Valentin L'Hôte[b], Erik Chorell[a,*], and Sjoerd Wanrooij[b,*]

Contents

Scheme S1 3
Supplementary Figure 1 4
Supplementary Figure 2 5
Supplementary Figure 3 6
Supplementary Figure 4 7
Supplementary Figure 5 8
Table S1: Oligonucleotides used in this study 9
Methods 10
Taq Polymerase STOP ASSAY 10
CELL CULTURE AND COMPOUNDS PREPARATION 10
CELL VIABILITY 11
LIVING CELLS IMAGING 11
MtDNA COPY NUMBER 11
PROTEIN EXTRACTION AND IMMUNOBLOT 12
CIRCULAR DICHROISM SPECTROSCOPY 12
FLUORESCENCE RESONANCE ENERGY TRANSFER ASSAY 12
NMR TITRATION ASSAY 13
General experimental 13
References 28
NMR Spectra 28

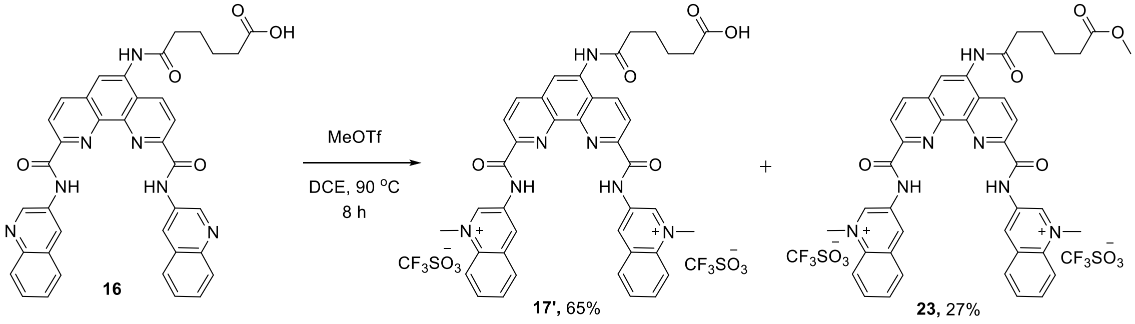

Scheme S1. Synthesis of Phen-DC3 linker reference compound **23**

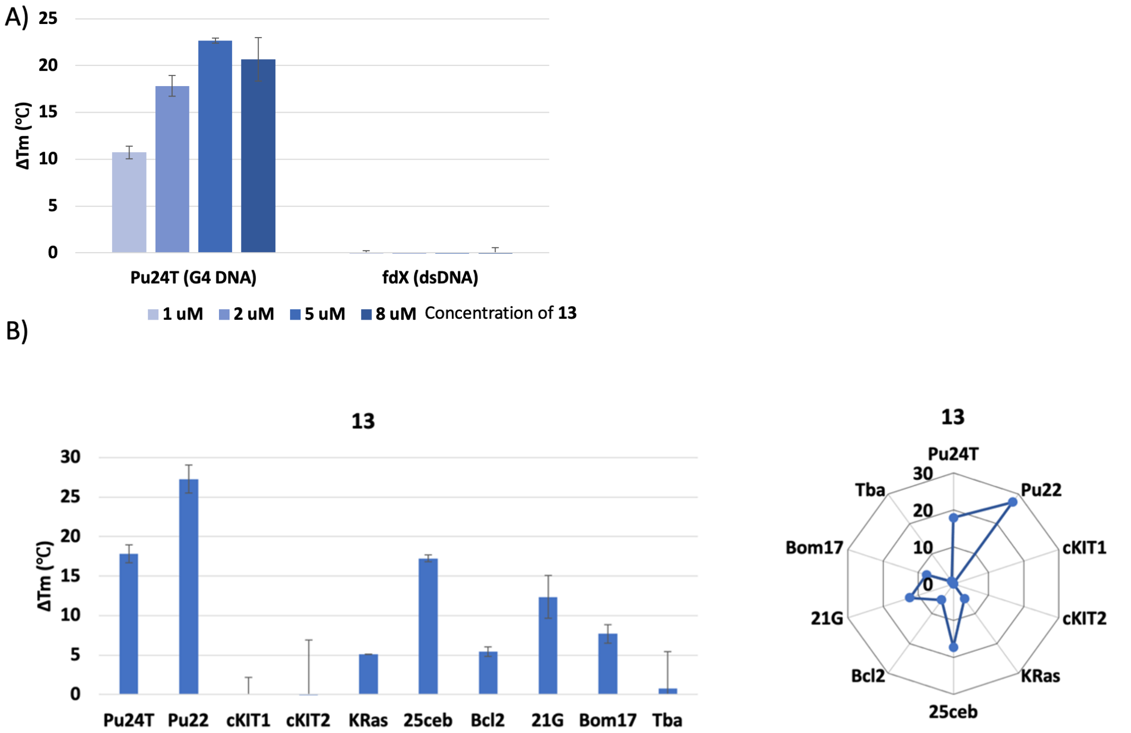

Supplementary Figure 1. Evaluation of the G4 stabilization and selectivity for BODIPY linked Phen-DC3 (**13**) using a fluorescence resonance energy transfer (FRET) assay. **A.** The stabilization effect of BODIPY linked Phen-DC3 (**13**) was evaluated at 1, 2, 5, and 8 μM on both the Pu24T G4 DNA (left) and the fdX dsDNA (right). **B.** The FRET melting assay with **13** at 2 μM concentrations for several G4 DNA structures (0.2 μM), showing the ability of the compounds to stabilise the different G4 DNA structures. Pu22 (*c-MYC* promoter); cKIt1, cKit2 (*c-KIT* promoter); KRas (*K-RAS* gene) and 25 ceb (human minisatellite) are parallel G4 forming sequences. Bcl2 (*BCL-2* promoter) and 21G (human telomere) are hybrid G4 forming sequences. Bom17 (Bombyx telomere) and Tba (thrombin binding aptamer) are antiparallel G4 forming sequences. Error bars correspond to SD of at least three independent experiments. T_m in the absence of ligands for Pu24T is 63.9 ± 0.5 °C; Pu22 is 59.4 ± 0.7 °C; c-KIT1 is 44.6 ± 1.4 °C; c-KIT2 is 69.4 ± 0.3 °C; KRas is 42.5 ± 0.7 °C; 25ceb is 72.4 ± 0.3 °C; Bcl2 is 63.0 ± 1.2 °C; 21G is 47.5 ± 1.0 °C; Bom17 is 44.2 ± 0.5 °C; and Tba is 41.8 ± 0.7 °C.

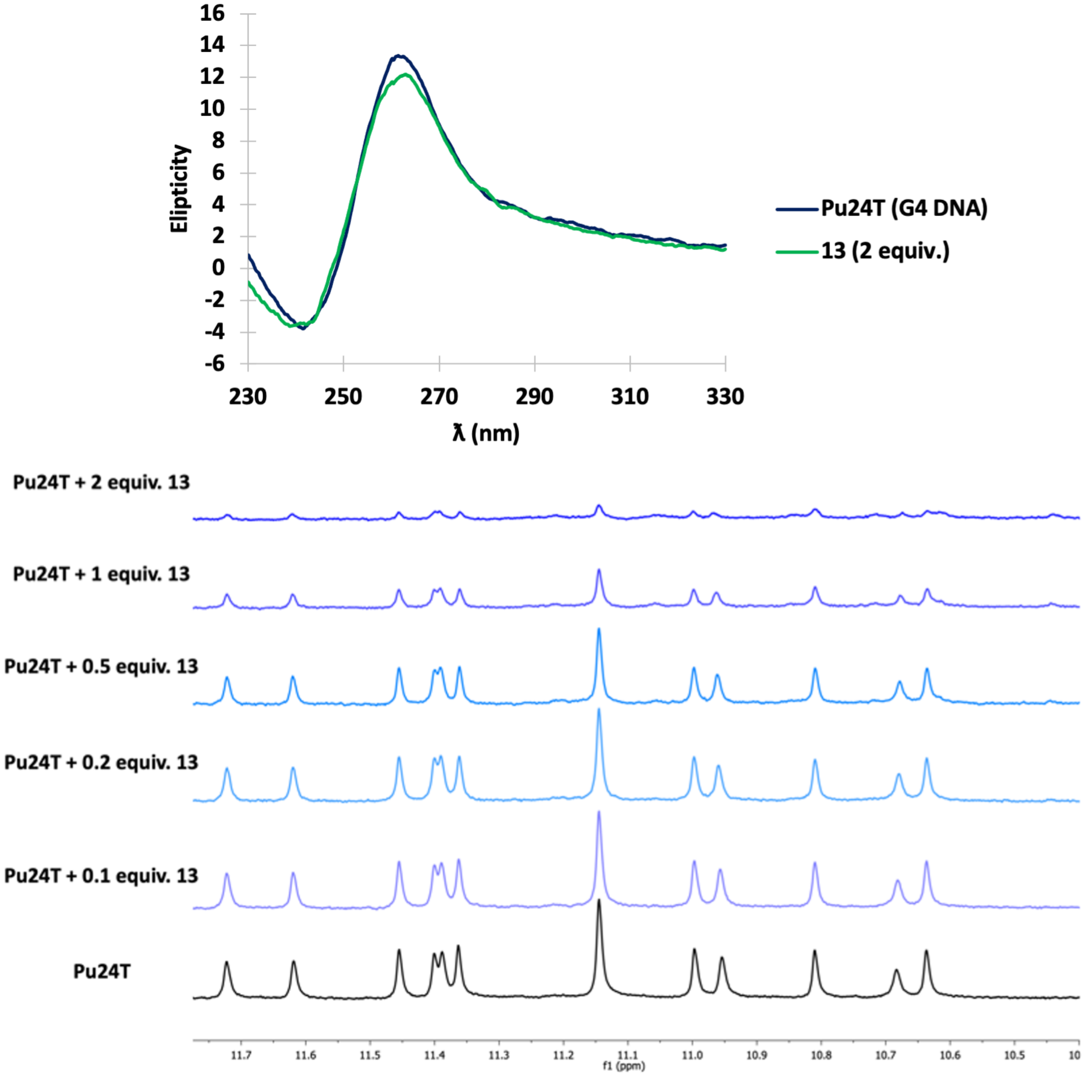

Supplementary Figure 2. **Top,** Circular dichroism (CD) spectroscopy of Pu24T G4 DNA alone and with 2 equivalents of BODIPY linked Phen-DC3 (**13**). **Bottom,** ^{1}H NMR (850 MHz) titrations for Pu24T with BODIPY linked Phen-DC3 (**13**). The initial DNA concentration was 90 µM and macrocycle was then added so the last addition corresponded to a total molar ratio of DNA:compound 1:2.

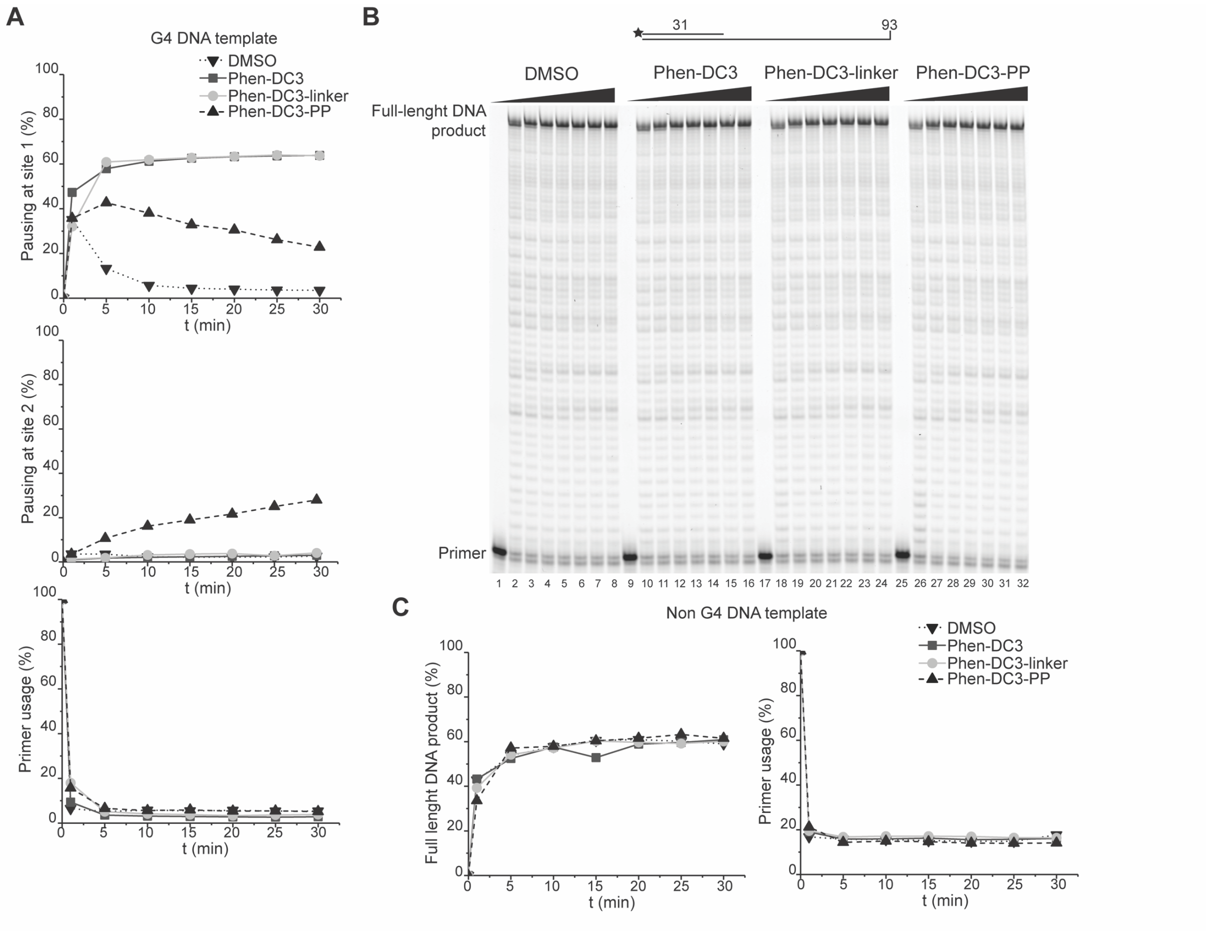

Supplementary Figure 3. **A.** Quantification of pausing site 1 and 2 and primer usage from gel in figure 4A. **B.** Taq-polymerase STOP assay on a NON G4 templates in the presence of 250 nM of the indicated compounds. For each compound, the reaction was blocked at increasing time points. **C.** Quantification of full-length DNA product (left) and primer usage (right) from gel in B. In A and C, values are represented as % of the total line signal.

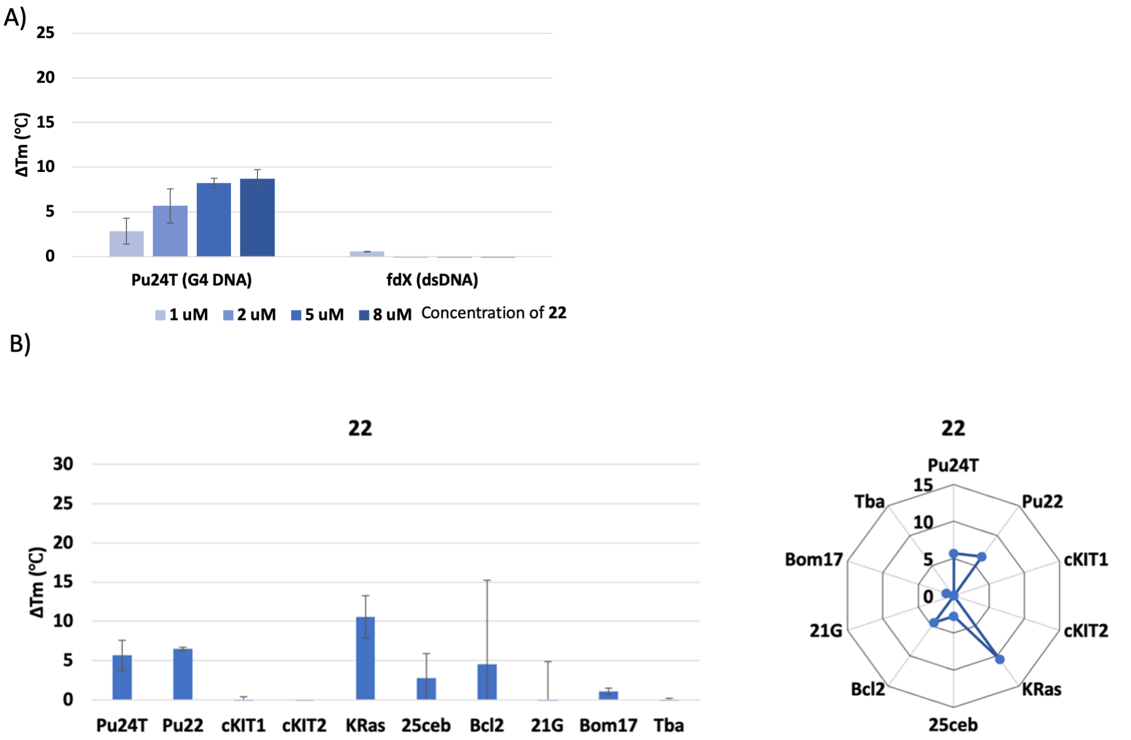

<u>Supplementary Figure 4.</u> Evaluation of the G4 stabilization and selectivity for Phen-DC3-PP (**22**) using a fluorescence resonance energy transfer (FRET) assay. **A.** The stabilization effect of Phen-DC3-PP (**22**) was evaluated at 1, 2, 5, and 8 µM on both the Pu24T G4 DNA (left) and the fdX dsDNA (right). **B.** The FRET melting assay with Phen-DC3-PP (**22**) at 2 µM concentrations for several G4 DNA structures (0.2 µM), showing the ability of the compounds to stabilise the different G4 DNA structures. Pu22 (*c-MYC* promoter); cKIt1, cKit2 (*c-KIT* promoter); KRas (*K-RAS* gene) and 25 ceb (human minisatellite) are parallel G4 forming sequences. Bcl2 (*BCL-2* promoter) and 21G (human telomere) are hybrid G4 forming sequences. Bom17 (Bombyx telomere) and Tba (thrombin binding aptamer) are antiparallel G4 forming sequences. Error bars correspond to SD of at least three independent experiments. T_m in the absence of ligands for Pu24T is 63.9 ± 0.5 °C; Pu22 is 59.4 ± 0.7 °C; c-KIT1 is 44.6 ± 1.4 °C; c-KIT2 is 69.4 ± 0.3 °C; KRas is 42.5 ± 0.7 °C; 25ceb is 72.4 ± 0.3 °C; Bcl2 is 63.0 ± 1.2 °C; 21G is 47.5 ± 1.0 °C; Bom17 is 44.2 ± 0.5 °C; and Tba is 41.8 ± 0.7 °C.

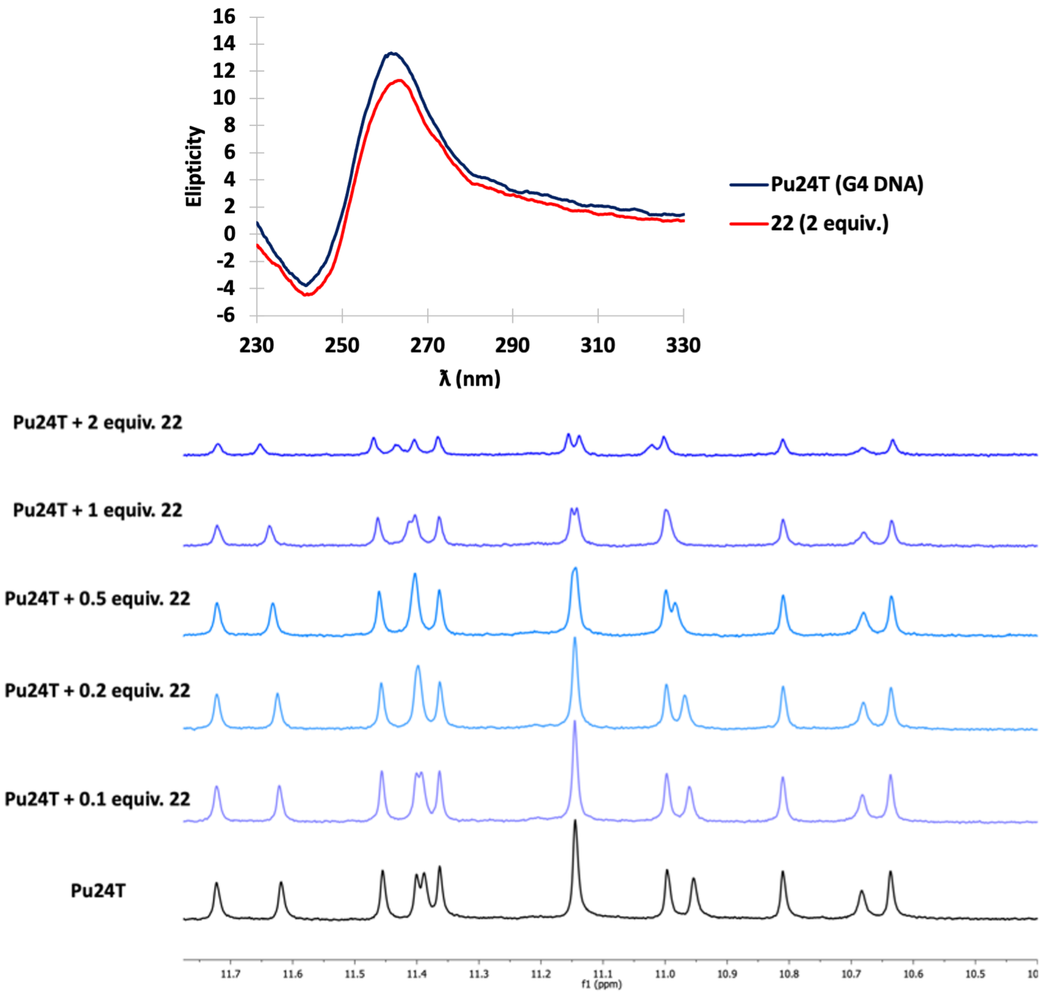

Supplementary Figure 5. **Top,** Circular dichroism (CD) spectroscopy of Pu24T G4 DNA alone and with 2 equivalents of Phen-DC3-PP (**22**). **Bottom,** ^{1}H NMR (850 MHz) titrations for *c-MYC* Pu24T with **22**. The initial DNA concentration was 90 µM and macrocycle was then added so the last addition corresponded to a total molar ratio of DNA:compound 1:2.

Table S1: Oligonucleotides used in this study

	Name	Sequence
**MtDNA copy number**		
MtDNA	ATP6 F	GCCGCAGTACTGATCATTC
	ATP6 R	CAGGTTCGTCCTTTAGTGTTG
nDNA	r18S F	ACGGACCAGAGCGAAAGCAT
	r18S R	GGACATCTAAGGGCATCACAGAC
**Taq Polymerase STOP assay**		
	Primer 5´-3´	TET-TGAAAACATTATTAATGGCGTCGAGCGTCCG
	c-MYCPu24T	ATATATATATTGAGGGTGGTGAGGGTGGGGAAGGATATATATAT CGGACGCTCGACGCCATTAATAATGTTTTCA
	Ribosomal G4	TGTTTTCCTTGGGGAAGGGTGGGGCATGTTATGGGAAGGTGGAG ATATATCGGACGCTCGACGCCATTAATAATGTTTTCA
	NonG4	GAGACCATTCAAAAGGATAATGTTTGTCATTTAGTATATGCCCCT GCTCGTCTTCCCTTCTCCGGACGCTCGACGCCATTAATAATGTTT TCA
**FRET assay**		
	FPu24TT	Fam-TGAG$_3$TG$_2$TGAG$_3$TG$_4$A$_2$G$_2$-Tamra
	FPu22T	Fam- TGAG$_3$TG$_3$TAG$_3$TG$_3$TA$_2$ -Tamra
	Fc-KIT1T	Fam-G$_3$AG$_3$CGCTG$_3$AG$_2$AG$_3$-Tamra
	Fc-KIT2T	Fam-G$_3$CG$_3$CGCGAG$_3$AG$_4$-Tamra
	FKrasT	Fam-AG$_3$CG$_2$TGTG$_3$AATAG$_3$AA-Tamra
	F25cebT	Fam- AG$_3$TG$_3$TGTAAGTGTG$_3$TG$_3$T –Tamra
	Fbom17T	Fam- G$_2$TTAG$_2$TTAG$_2$TTG$_2$ -Tamra
	FBcl2T	Fam-G$_3$CGCG$_3$AG$_2$AATTG$_3$CG$_3$-Tamra
	F21GT	Fam- G$_3$TTAG$_3$TTAG$_3$TTAG$_3$ -Tamra
	FtbaT	Fam- G$_2$TTG$_2$TGT G$_2$TTG-Tamra
	Fds26T	Fam-CAATCGGATCGAATTCGATCCGATTG-Tamra (competitor DNA)
	ds26	CAATCGGATCGAATTCGATCCGATTG

Methods

Taq Polymerase STOP ASSAY

Taq Polymerase STOP assays were performed as previously described.[1] Briefly, 40 nM of annealed templates (Table S1) were mixed with the different compounds at the indicated concentrations in a reaction buffer containing 10 mM of Tris pH 8, 50 mM of KCl, 0,2 µM of dNTPs, 1.5 mM of $MgCl_2$. Final DMSO concentration was 1%. The reaction was initiated by the addition of the Taq DNA Polymerase (Thermo Fisher Scientific) at a final concentration of 0.0625 U/µL and carried out at 50 °C for up to 30′. Samples were separated on a denaturing 10% polyacrylamide Tris-Borate-EDTA (TBE) gel containing 25% of formamide and 8 M of urea. Fluorescent signal was detected with Typhoon 9400 scanner (Amersham Biosciences). The intensity of each band was quantified in each lane as a percentage of total lane signal using Image Quant TL 8.1 (GE Healthcare Life Sciences).

CELL CULTURE AND COMPOUNDS PREPARATION

HeLa cells (epithelioid cervix carcinoma, purchased from Sigma-Aldrich) were routinely cultured at 37 °C - 7% CO_2 in DMEM high glucose medium with Glutamax (Gibco) supplemented with 1 mM Sodium-pyruvate, penicillin-streptomycin and 10% fetal bovine serum (FBS). Cells were tested for absence of mycoplasma.

Compounds were dissolved in DMSO to 50 mM (Phen-DC3 and BODIPY) or 20 mM (Phen-DC3-BODIPY, Phen-DC3-linker, Phen-DC3-PP and PP) aliquoted and stored at -20 °C. Prior to addition to cells, compounds were dissolved in the medium at the final concentrations required.

CELL VIABILITY

Cell viability was measured using PrestoBlue cell viability reagent (Invitrogen) according to manufacturer's recommendations. Briefly, 5000 cells/well were seeded in the indicated medium on 96 wells plate the day before treatment. Compounds were dissolved in medium at the indicated concentrations and added to cells. 48 hours after treatment, 10 µl of PrestoBlue was added each well and cells were incubated at 37 °C for three additional hours. Fluorescence (Ex.560-Em.590, 10nm bandwidth) was recorded using Synergy H4 microplate reader (Biotek).

LIVING CELLS IMAGING

200.000 cells/well were seeded the day before treatment in 6 well plates. Cells were treated at the indicated concentrations for 12 h, trypsinized and let them seeded on the bottom of glass plates for around 6h. Before imaging, cells were treated with MitoTracker Red CMXRos 0,2 µg/ml (Thermo Fisher Scientific) and Hoechst 33342 5µg/ml (Thermo Fisher Scientific). Imaging was performed at 37 °C, 5% CO_2 with NIKON A1R Confocal equipped with DIC 60X oil objective with laser line 405 nm (for Hoestch), 488nm (for BODIPY) and 561 nm (for Mitotracker). Images were processed using ImageJ software.

MtDNA COPY NUMBER

MtDNA copy number was measured by quantitative real time PCR (qPCR). 200.000 cells (for 12h treatment) or 100.000 cells (for 24h treatment)/well were seeded the day before treatment. After treatment, cells were collected by trypsinization and total genomic DNA was extracted using the PureLink Genomic DNA Minikit (Thermo Fisher Scientific). Each DNA sample was diluted to 10ng/µl and 2µl were used for amplification using qPCRBIO SyGreen Mix (PCR Biosystem) with LightCycler96 (Roche) instrument. Oligonucleotides for nuclear and mitochondrial DNA amplification are indicated in table S1. Each sample

was run in triplicate. Mean Ct values were used to calculate the mtDNA copy number relative to untreated samples using ΔΔCt method.

PROTEIN EXTRACTION AND IMMUNOBLOT

HeLa cells were seeded on 10 cm dishes the day before treatment in order to have 80-90% confluency the day after. Cells were treated for 12 h with the indicated concentrations. Nuclear histones-bound protein fractions were extracted as previously described.[2] Equal amounts (10 µg) of proteins were separated on 4-20% SDS-TGX (Bio-Rad) gels and transferred to a 0,45 µM nitrocellulose membrane (GE Healthcare Life Sciences) using Mini-protean electrophoresis System (Bio-Rad). Membranes were blocked in 5% non-fat milk for 2 h. Primary antibodies (Cell Signalling, H2B #12364 and γH2A.X #2577) diluted 1:1000 were incubated overnight at 4 °C. Horseradish Peroxidase (HRP) conjugated goat anti rabbit secondary antibody (ThermoScientific) diluted 1:3000 were incubated 1h at room temperature. All washes and incubations were performed in Tris-buffered saline Tween 20 (T-TBS) buffer. Antibodies were diluted in 5% non-fat milk. Chemiluminescent detection was performed using ECL western blotting substrates (ThermoScientific) and ChemiDoc Touch Imaging System (Bio-Rad). Signal quantification was performed using ImageQuant TL Software (GE Healthcare Life Sciences).

CIRCULAR DICHROISM SPECTROSCOPY

5 µM of Pu24T G4-DNA was folded in 10 mM K-phosphate buffer (pH 7.4), with 5 mM KCl by heating for 5 min at 95 °C and then allowed for cooling to room temperature. A quartz cuvette with a path length of 1 mm was used for the measurements by JASCO-720 spectropolarimeter (Jasco Internatiol Co. Ltd.). CD spectra were recorded at 25 °C over λ = 230-350 nm with an interval of 0.2 nm and a scan rate of 100 nm/min.

FLUORESCENCE RESONANCE ENERGY TRANSFER ASSAY

The fluorescence resonance energy transfer (FRET) occurs between two dyes (5'-FAM as donor and 3'- TAMRA as acceptor) linked at both extremities of a DNA oligonucleotide.

When the oligonucleotides are folded into G4 structures, the donor and acceptor are in close proximity, which results in an energy transfer from the donor to the acceptor. This process can be detected by a reduction in the fluorescence emission of the donor. Fluorescence emission of the donor is recovered when the temperature increment triggers the thermal denaturation of the G4 structure. The experiments were performed in a Bio-rad CFX96 real-time PCR device at temperatures from 10 to 95 °C at 1.5 °C/min heating rate using a 492-nm excitation wavelength and a 516-nm detection wavelength in 96-well plates. Each condition was tested in duplicate and analysis of the data was carried out by using Excel and Origin 8 software. In each well, 0.2 μM of labelled oligonucleotide was heated in the presence or absence of the ligand at the specified concentrations. Emission of 5'-FAM was normalized between 0 and 1, and the melting temperature (Tm) is defined as the temperature at which 50% of the G4 structures are denatured (the temperature when the normalized emission was 0.5). The stabilization (ΔTm) is calculated from comparison of Tm of the fluorescently labelled oligonucleotide in the presence or absence of the ligand.

NMR TITRATION ASSAY

The G4 DNA stock solution was prepared by folding 100 μM *c-MYC* Pu24T in 10 mM potassium phosphate buffer (pH = 7.4) and 35 mM KCl by heating to 95 °C and cooling to ambient temperature. 10% D_2O was added to the DNA stock solution, yielding a final DNA concentration of 90 μM. NMR samples were prepared by the sequential addition of **13** or **22** from 2 mM or 10 mM DMSO stock solutions to 200 μL of the DNA solution in 3 mm NMR tubes. All spectra were recorded at 298 K on a Bruker 850 MHz Avance III HD spectrometer equipped with a 5 mm TCI cryoprobe. Excitation sculpting was used in the 1D 1H experiments, and 256 scans were recorded. Processing of spectra was performed in MestreNova 10.0.2.

General experimental

All reagents and solvents were used as received from commercial suppliers unless stated otherwise. TLC was performed on aluminum backed silica gel plates (median pore size 60 Å, fluorescent indicator 254 nm) and detected with UV light. Flash column

chromatography was performed using silica gel with an average particle diameter of 50 μm (range 40−65 μm, pore diameter 53 Å), eluents are given in brackets. DMF, THF and DCM were dried in a solvent drying system (THF and DCM drying agent: neutral alumina; DMF drying agent: activated molecular sieves, also equipped with an isocyanate scrubber) and were collected fresh prior to every reaction. ^{1}H and ^{13}C NMR spectra were recorded on a Bruker 400 MHz spectrometer at 298 K or on a Bruker 600 MHz spectrometer at 298 K, and calibrated by using the residual peak of the solvents as the internal standard ($CDCl_3$: δ H = 7.26 ppm; δ C = 77.16 ppm. DMSO-d_6: δ H = 2.50 ppm; δ C = 39.50 ppm). LC-MS was conducted on an Agilent 6150 Series Quadrupole LC/MS system. Preparatory HPLC was performed on a C18 reversed-phase column (25 cm x 21.2 mm, 5 mm) with H2O/MeCN mixtures as the eluent. Solid phase synthesis of the peptide was carried out on SyroI peptide synthesizer.

Abbreviations: DMF – dimethylformamide, MeOH – methanol, THF – tetrahydrofuran, rt – room temperature, EtOAc – ethyl acetate, DCE – dichloroethane, TFA – trifluoro acetic acid, DCM – dichloromethane, NaH – sodium hydride, MeCN – acetonitrile, TLC – thin layer chromatography, LC-MS – liquid chromatography mass spectrometry, Pd/C – palladium on carbon, , HATU – 1-[Bis(dimethylamino)-methylene]-1H-1,2,3-triazolo[4,5-b]pyridinium3-oxide, DIPEA – *N*,*N*-Diisopropylethyl-amine, HPLC – high pressure liquid chromatography.

Procedure for preparation of 2,9-dimethyl-5-nitro-1,10-phenanthroline (2)

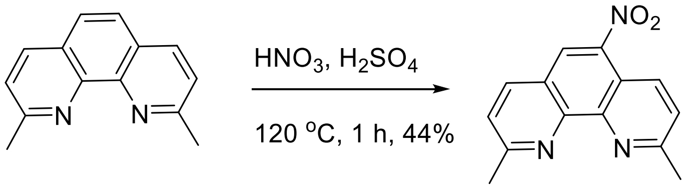

The cool mixture of nitric acid (HNO_3) (5 ml) and fumed sulfuric acid (H_2SO_4) (10 ml) was added drop-wise to 2,9-dimethyl-1,10-phenanthroline (5 g, 24 mmol) in fumed sulfuric acid (5 mL) at 0 °C. Then, the reaction mixture was stirred at 120 °C for 3 h. The progress of the reaction was monitored by TLC. Upon completion, the reaction mixture was poured

into crushed ice (20 grams) and neutralized with 2M NaOH solution until pH 8. A yellow precipitate was formed that was isolated by filtration. The solid was dissolved in EtOAc and washed with water and dried under vacuum to give product 6-nitroindoline[3] **2** as a yellow solid in 44% yield.

^{1}H NMR (600 MHz, DMSO-d_6): δ = 8.91 (s, 1H), 8.77 (d, J = 8.6 Hz, 1H), 8.62 (d, J = 8.2 Hz, 1H), 7.81 (d, J = 8.9 Hz, 1H), 7.79 (d, J = 8.3 Hz, 1H), 2.85 (s, 3H), 2.84 (s, 3H); ^{13}C NMR (150 MHz, DMSO-d_6): δ 162.93, 160.30, 146.64, 145.19, 143.60, 138.88, 132.52, 125.35, 125.23, 125.15, 124.13, 118.99, 25.91, 25.39; MS (ES mass): m/z 587.3 (M+1).

Procedure for preparation 5-nitro-1,10-phenanthroline-2,9-dicarbaldehyde (3)

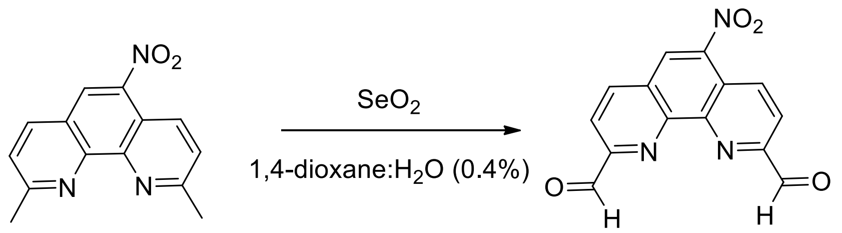

To a solution of 2,9-dimethyl-5-nitro-1,10-phenanthroline **2** (5 g, 19.7 mmol) in 0.4% H_2O and 1,4-dioxane (20 mL) was added SeO_2 (5 g, 45.06 mmol) at room temperature under nitrogen atmosphere. The reaction mixture was stirred at reflux for 3 h. The progress of the reaction was monitored by TLC. Upon completion, the reaction mixture was filtered through the celite pad and the filtrate was concentrated under reduced pressure. The crude compound was used for the next step without purification.

Procedure for preparation 2,9-dimethyl-5-nitro-1,10-phenanthroline (4)

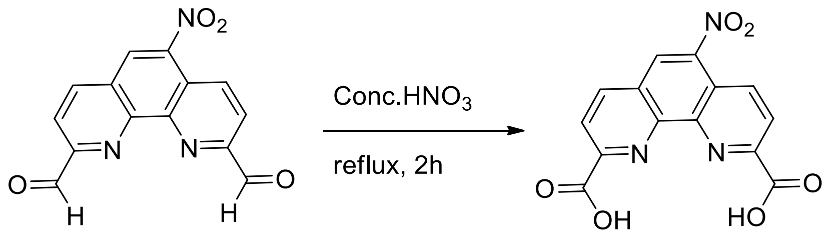

The 5-nitro-1,10-phenanthroline-2,9-dicarbaldehyde **2** (5 g, 17.7 mmol) dissolved in concentrated HNO_3 at room temperature. The reaction mixture was stirred at reflux for 2

h. The progress of the reaction was monitored by TLC. Upon completion, the reaction mixture was poured into crushed ice (20 grams) which resulted in the formation of a yellow-colored precipitate that was isolated by filtration. The solid was washed with water and dried under vacuum to give product 5-nitro-1,10-phenanthroline-2,9-dicarboxylic acid[3] **4** as a yellow solid in 56% yield (over 2 steps).

^{1}H NMR (600 MHz, DMSO-d_6): δ = 13.84 (s, 2H), 9.22 (s, 1H), 9.08 (d, *J* = 8.7 Hz, 1H), 8.99 (d, *J* = 8.3 Hz, 1H), 8.54 (d, *J* = 8.7 Hz, 1H), 8.51 (d, *J* = 8.2 Hz, 1H); ^{13}C NMR (150 MHz, DMSO): δ = 166.49, 166.31, 151.92, 149.92, 146.74, 145.61, 145.29, 140.89, 134.69, 128.06, 127.70, 124.87 (2C), 122.76; MS (ES mass): *m*/*z* 314.2 (*M*+1).

Procedure for preparation 5-nitro-*N*^{2},*N*^{9}-di(quinolin-3-yl)-1,10-phenanthroline-2,9-dicarboxamide (5)

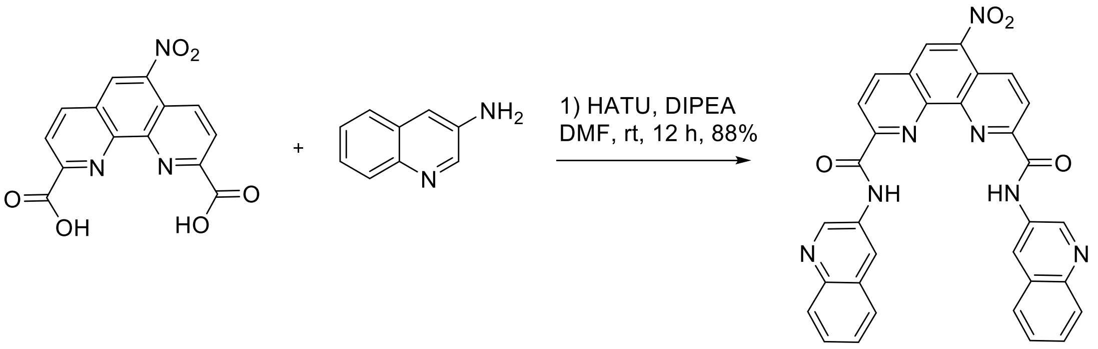

To the solution of 5-nitro-1,10-phenanthroline-2,9-dicarboxylic acid **4** (0.50 g, 1.59 mmol) in a dry DMF (10 mL) was added HATU (1.82 g, 4.78 mmol) and the reaction mixture was stirred until the solids were dissolved completely at room temperature. Then the quinoline-3-amine **7** (0.46 g, 3.19 mmol) in DMF (5 mL) was slowly added to the reaction mixture and stirred for 10 min followed by the addition of DIPEA (0.62 g, 4.78 mmol). The mixture was stirred at room temperature under nitrogen atmosphere for 12 h. The progress of the reaction was monitored by TLC. Upon completion, the reaction mixture was poured into ice and filtered through sintered funnel. The solid was washed with DCM and dried under vacuum. The pure 5-nitro-*N*^{2},*N*^{9}-di(quinolin-3-yl)-1,10-phenanthroline-2,9-dicarboxamide **5** was isolated in 88% yield as a light yellow solid.

^{1}H NMR (600 MHz, DMSO-d_6): δ = 11.82 (s, 1H), 11.78 (s, 1H), 9.64 (s, 2H), 9.24 (s, 1H), 9.17 (d, *J* = 8.7 Hz, 1H), 9.07 (d, *J* = 9.5 Hz, 3H), 8.73 (dd, *J* = 11.9, 8.7 Hz, 2H),

8.07 (d, J = 8.2 Hz, 4H), 7.75 (t, J = 7.7 Hz, 2H), 7.66 (t, J = 7.6 Hz, 2H); ^{13}C NMR (150 MHz, DMSO): δ = 163.28, 163.17, 152.55, 150.62, 145.75 (3C), 145.66, 145.36, 144.95 (2C), 144.49, 141.60, 135.49, 132.71 (2C), 128.99 (3C), 128.55 (3C), 128.22, 127.79 (2C), 127.71 (3C), 124.55 (2C), 123.24 (2C); MS (ES mass): *m*/*z* 566.3 (*M*+1).

Procedure for preparation 5-amino-*N*²,*N*⁹-di(quinolin-3-yl)-1,10-phenanthroline-2,9-dicarboxamide (6)

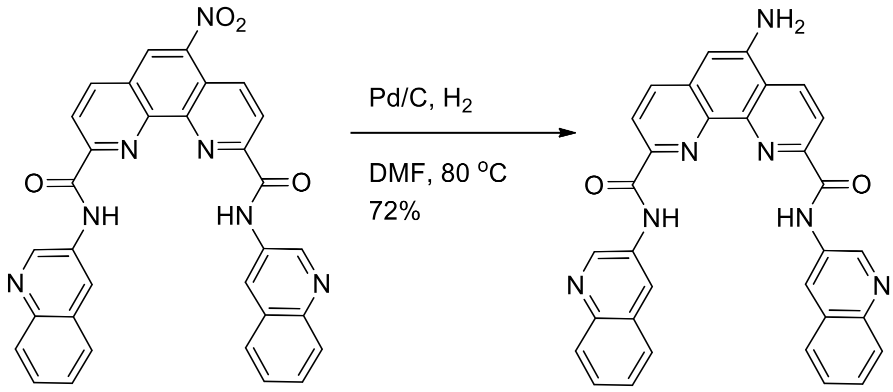

To a solution of 5-nitro-N^2,N^9-di(quinolin-3-yl)-1,10-phenanthroline-2,9-dicarboxamide (5 g, 8.85 mmol) in DMF (35 mL) was added (10 mol%) 10% Pd/C and the reaction mixture was stirred under hydrogen atmosphere at room temperature for 3 h. The progress of the reaction was monitored by TLC. Upon completion, the reaction mixture was filtered through a celite pad and the filtrate was concentrated under reduced pressure. The crude 5-amino-N^2,N^9-di(quinolin-3-yl)-1,10-phenanthroline-2,9-dicarboxamide **6** was isolated in 72% yield as light greenish solid and was used for further reaction without any purification. ^{1}H NMR (400 MHz, DMSO-d_6): δ = 11.85 (s, 1H), 11.69 (s, 1H), 9.69 (d, J = 2.5 Hz, 1H), 9.66 (d, J = 2.5 Hz, 1H), 9.13 (dd, J = 9.9, 2.5 Hz, 2H), 9.09 (d, J = 8.6 Hz, 1H), 8.64 (d, J = 8.5 Hz, 1H), 8.41 (d, J = 2.1 Hz, 2H), 8.13 – 8.06 (m, 4H), 7.79 – 7.71 (m, 2H), 7.70 – 7.64 (m, 2H), 7.12 (s, 1H), 6.78 (s, 2H); ^{13}C NMR (150 MHz, DMSO): δ = 163.22, 163.11, 152.52, 150.57, 145.79, 145.77, 145.70, 145.60, 145.25, 145.03 (2C), 144.43, 141.51, 135.36, 132.67, 129.06 (3C), 128.87, 128.52 (3C), 128.43, 128.18, 128.16, 127.75, 127.63 (3C), 124.36, 123.18, 123.11; MS (ES mass): *m*/*z* 536.3 (*M*+1).

Procedure for preparation 5-(2-bromoacetamido)-N^2,N^9-di(18uinoline-3-yl)-1,10-phenanthroline-2,9-dicarboxamide (8)

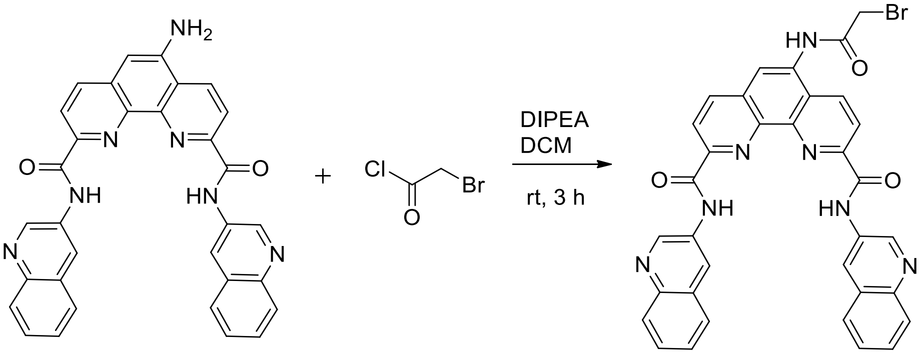

To a stirred solution of 5-amino-N^2,N^9-di(18 uinoline-3-yl)-1,10-phenanthroline-2,9-dicarboxamide **6** (0.5 g, 0.93 mmol) in dry DCM (10 mL) was added 2-bromoacetyl chloride (0.28 g, 1.4 mmol) and *N*,*N*-Diisopropylethylamine (DIPEA) (0.18 g, 1.4 mmol) at room temperature. Then the mixture was stirred at room temperature under nitrogen atmosphere for 3 h. The progress of the reaction was monitored by TLC. Upon completion, the reaction mixture was filtered through sintered funnel. The solid was washed with DCM and diethyl ether then dried under vacuum to give 5-(2-bromoacetamido)-N^2,N^9-di(quinolin-3-yl)-1,10-phenanthroline-2,9-dicarboxamide **8** in 80% yield as brown solid. ^{1}H NMR (600 MHz, DMSO-d_6): δ = 11.95 (s, 1H), 11.94 (s, 1H), 10.80 (s, 1H), 9.82 (d, *J* = 2.4 Hz, 2H), 9.34 (d, *J* = 8.4 Hz, 2H), 9.03 (d, *J* = 8.6 Hz, 1H), 8.86 (d, *J* = 8.4 Hz, 1H), 8.72 (d, *J* = 8.5 Hz, 1H), 8.63 (d, *J* = 8.2 Hz, 1H), 8.51 (s, 1H), 8.22 (d, *J* = 8.1 Hz, 2H), 8.16 (d, *J* = 8.4 Hz, 2H), 7.89 – 7.86 (m, 2H), 7.80 – 7.77 (m, 2H), 4.62 (s, 2H); ^{13}C NMR (150 MHz, DMSO): δ = 169.05, 166.81, 163.82, 163.63, 149.50, 148.96, 144.59, 144.19, 143.97, 142.46, 138.96, 134.82, 133.42 (2C), 133.15 (2C), 133.05 (2C), 131.11, 130.44, 128.85 (3C), 128.60, 128.51, 128.48 (2C), 127.57, 127.32, 126.54, 122.50, 122.17, 121.81, 43.89; MS (ES mass): *m*/*z* 656.2 (*M*+1).

Procedure for preparation 5-(2-azidoacetamido)-*N*^{2},*N*^{9}-di(quinolin-3-yl)-1,10-phenanthroline-2,9-dicarboxamide (9)

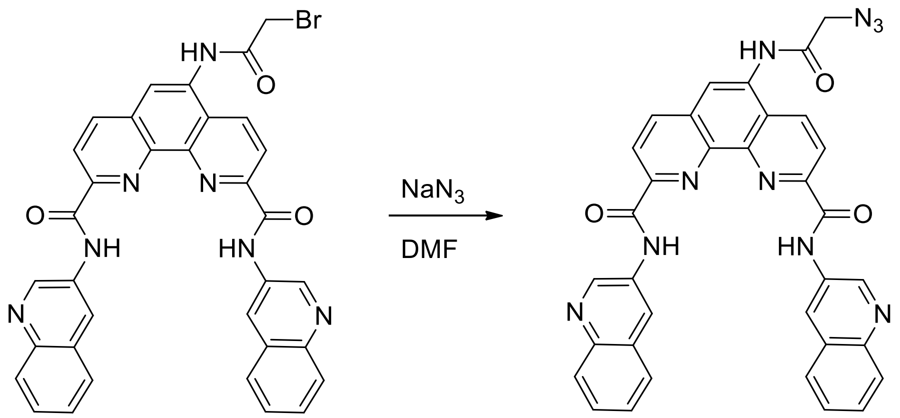

To a stirred solution of 5-(2-bromoacetamido)-*N*^{2},*N*^{9}-di(quinolin-3-yl)-1,10-phenanthroline-2,9-dicarboxamide **8** (0.2 g, 0.304 mmol) in dry DMF (10 mL) was added sodium azide (0.03 g, 0.45 mmol) at room temperature. The mixture was stirred at room temperature under nitrogen atmosphere for 12 h. The progress of the reaction was monitored by TLC. Upon completion, the solvent was removed under reduced pressure and diethyl ether was added to the crude. The resulting solid was filtered through sintered funnel. The solid was washed with DCM and diethyl ether then dried under vacuum to give 5-(2-azidoacetamido)-*N*^{2},*N*^{9}-di(quinolin-3-yl)-1,10-phen-anthroline-2,9-dicarboxamide **9** in 70% yield as brown solid.

^{1}H NMR (600 MHz, DMSO-*d*$_6$): δ = 11.85 (s, 1H), 11.82 (s, 1H), 10.62 (s, 1H), 9.69 (dd, *J* = 8.7, 2.4 Hz, 2H), 9.14 (dd, *J* = 6.3, 2.4 Hz, 2H), 9.04 (d, *J* = 8.5 Hz, 1H), 8.86 (d, *J* = 8.2 Hz, 1H), 8.72 (d, *J* = 8.5 Hz, 1H), 8.63 (d, *J* = 8.2 Hz, 1H), 8.55 (s, 1H), 8.11 – 8.08 (m, 4H), 7.77 – 7.74 (m, 2H), 7.69 – 7.66 (m, 2H), 4.39 (s, 2H); ^{13}C NMR (150 MHz, DMSO): δ = 168.45, 163.77, 163.59, 149.82, 149.25, 145.89, 145.84, 145.04, 144.97, 144.63, 142.39, 138.80, 134.70, 133.26, 132.89, 132.82, 131.04, 129.10, 129.08, 128.84, 128.77, 128.51, 128.49, 128.28, 128.25, 127.65, 127.64, 127.63, 127.10, 124.30, 124.10, 122.44, 121.70, 51.79; MS (ES mass): *m*/*z* 619.2 (*M*+1).

Procedure for preparation 5-(2-aminoacetamido)-*N*^{2},*N*^{9}-di(quinolin-3-yl)-1,10-phenanthroline-2,9-dicarboxamide (10)

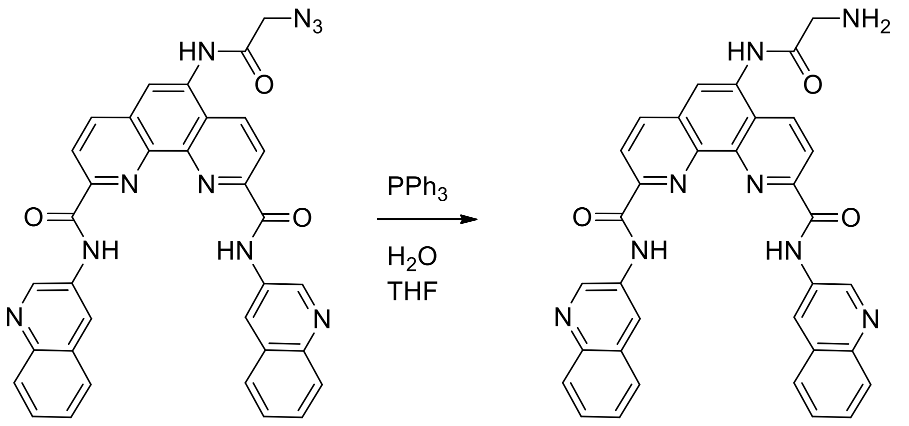

To a stirred solution of 5-(2-azidoacetamido)-N^2,N^9-di(quinolin-3-yl)-1,10-phen-anthroline-2,9-dicarboxamide **9** (0.15 g, 0.24 mmol) in THF (10 mL) and H_2O (5 mL) was added triphenylphosphine (0.12 g, 0.48 mmol) at room temperature. The mixture was stirred at 90 °C under nitrogen atmosphere for 8 h. The progress of the reaction was monitored by TLC. Upon completion, the solvent was removed under reduced pressure. Water was added to the crude and the resulting slurry was filtered through sintered funnel. Diethyl ether was added to the obtained solid in flask and filtered through sintered funnel and this process was continued for three times. The solid was washed with DCM and diethyl ether then dried under vacuum to give 5-(2-azidoacetamido)-N^2,N^9-di(quinolin-3-yl)-1,10-phen-anthroline-2,9-dicarboxamide **9** in 70% yield as brown solid.

^{1}H NMR (400 MHz, DMSO-d_6): δ = 11.86 (s, 1H), 11.82 (s, 1H), 9.70 – 9.68 (m, 2H), 9.14 (s, 2H), 8.97 (d, J = 8.6 Hz, 1H), 8.84 (d, J = 8.3 Hz, 1H), 8.73 (d, J = 8.5 Hz, 1H), 8.62 (d, J = 10.5 Hz, 2H), 8.10 (d, J = 8.2 Hz, 5H), 7.77 – 7.68 (m, 6H), 3.63 (s, 2H); ^{13}C NMR (150 MHz, DMSO): δ = 163.81, 163.60, 149.80, 149.00, 145.94, 145.89, 145.11, 145.05, 144.66, 142.15, 138.65, 134.23, 133.64, 132.90, 132.82, 131.97, 131.24, 129.16 (2C), 128.82, 128.74, 128.51, 128.48, 128.28, 128.25, 127.64, 127.62, 126.87, 124.25, 124.01, 121.80, 122.43, 120.02, 45.44; MS (ES mass): m/z 593.2 (M+1).

Procedure for preparation 5-(2-(3-(5,5-difluoro-1,3,7,9-tetramethyl-5H-4λ^4,5λ^4-di-pyrrolo[1,2-c:2',1'-*f*][1,3,2]diazaborinin-10-yl)propanamido)acetamido)-*N*^{2},*N*^{9}-di(quino-lin-3-yl)-1,10-phenanthroline-2,9-dicarboxamide (12)

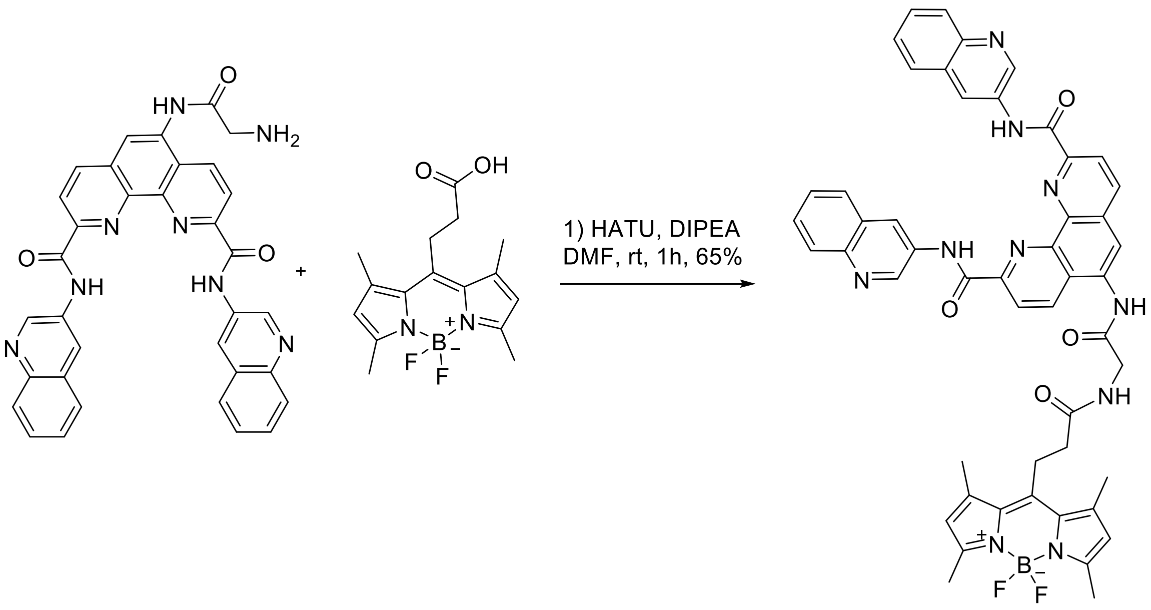

To the solution of 5-(2-aminoacetamido)-N[2],N[9]-di(quinolin-3-yl)-1,10-phenanthroline-2,9-dicarboxamide **10** (0.2 g, 0.33 mmol) in a dry DMF (6 mL) was added HATU (0.13 g, 0.35 mmol) and the reaction mixture was stirred until the solids were dissolved completely at room temperature. Then the 3-(5,5-difluoro-1,3,7,9-tetramethyl-5H-4λ^4,5λ^4-dipyrrolo[1,2-c:2',1'-f][1,3,2]diazaborinin-10-yl)propanoic acid **11**[4] (0.11 g, 0.33 mmol) in DMF (2 mL) was slowly added to the reaction mixture and stirred for 10 min followed by the addition of DIPEA (0.66 g, 0.51 mmol). The mixture was stirred at room temperature under nitrogen atmosphere for 12 h. The progress of the reaction was monitored by TLC. Upon completion, the reaction mixture was poured into ice and filtered through a sintered funnel. The crude was purified on silica gel using DCM and MeOH as a eluent to give 5-(2-(3-(5,5-difluoro-1,3,7,9-tetramethyl-5H-4λ^4,5λ^4-dipyrrolo[1,2-c:2',1'-*f*][1,3,2]diaza-borinin-10-yl)propanamido)acetamido)-N^2,N^9-di(quinolin-3-yl)-1,10-phenanthroline-2,9-dicarboxamide **12** in 65% yield as a light orange solid.

^{1}H NMR (600 MHz, DMSO-d_6): δ = 11.86 (s, 1H), 11.83 (s, 1H), 10.49 (s, 1H), 9.70 (d, *J* = 2.5 Hz, 1H), 9.68 (d, *J* = 2.5 Hz, 1H), 9.14 (s, 2H), 9.04 (d, *J* = 8.5 Hz, 1H), 8.85 (d, *J* = 8.2 Hz, 1H), 8.71 (d, *J* = 8.4 Hz, 1H), 8.66 – 8.60 (m, 2H), 8.50 (s, 1H), 8.11 – 8.09 (m, 4H), 7.75 (q, *J* = 6.7 Hz, 2H), 7.68 (td, *J* = 12.1, 5.7 Hz, 2H), 6.27 (s, 2H), 4.25 (d, *J* = 5.8 Hz, 2H), 3.32 – 3.28 (m, 2H), 2.60 – 2.58 (m, 2H), 2.52 (s, 6H), 2.42 (s, 6H); ^{13}C NMR (150 MHz, DMSO): δ = 171.42, 169.72, 163.80, 163.62, 153.92, 149.80, 149.17, 146.07, 145.93 (2C), 145.89, 145.11, 145.05, 144.65, 142.34, 141.55 (2C), 138.74, 134.82, 133.76,

132.90, 132.83, 131.13, 131.11, 129.15 (3C), 128.81, 128.74, 128.51, 128.49, 128.28, 128.26, 127.64, 127.62, 127.31, 124.23, 124.02, 122.42, 122.25 (3C), 121.61, 43.40, 36.52, 24.35, 16.54 (3C), 14.59; MS (ES mass): *m*/*z* 895.2 (*M*+1).

Procedure for preparation of 3,3'-((5-(3-(3-(5,5-difluoro-1,3,7,9-tetramethyl-5H-4λ^4,5 λ^4-dipyrrolo[1,2-c:2',1'-f][1,3,2]diazaborinin-10-yl)propanamido)propanamido)-1,10-phenanthroline-2,9-dicarbonyl)bis(azanediyl))bis(1-methylquinolin-1-ium) diiodide (13)

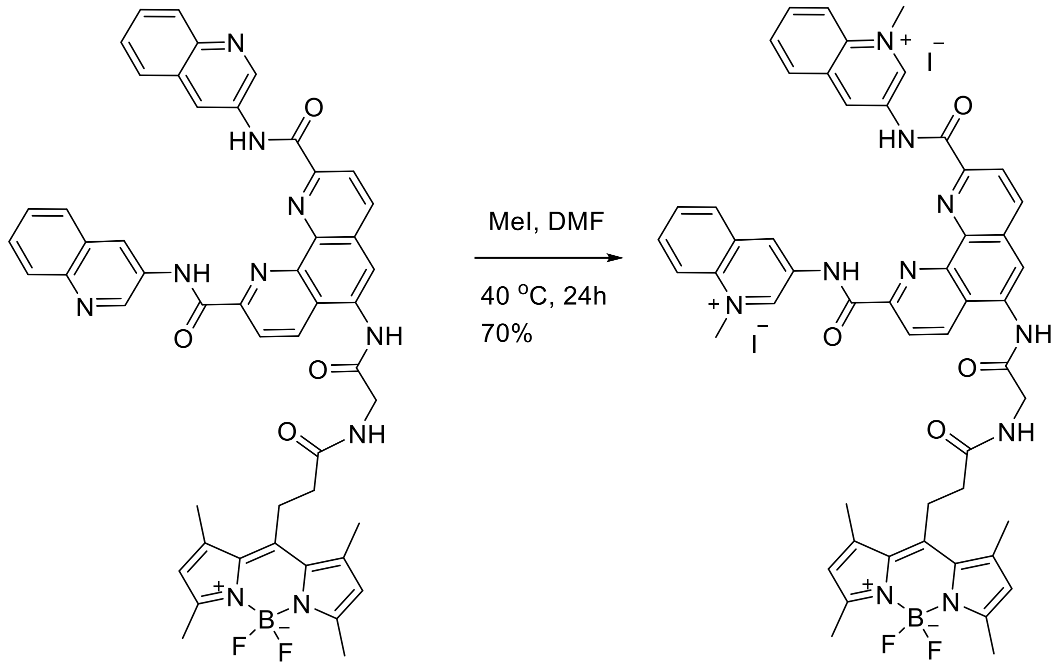

To the solution of 5-(3-(3-(5,5-difluoro-1,3,7,9-tetramethyl-5*H*-4λ^4,5λ^4-dipyrrolo[1,2-c:2',1'-*f*][1,3,2]diazaborinin-10-yl)propanamido)propanamido)-*N*^{2},*N*^{9}-di(quinolin-3-yl)-1,10-phenanthroline-2,9-dicarboxamide (0.05 g, 0.05 mmol) in dry DMF (5 mL) was added methyl iodide (0.12 g, 0.82 mmol) at room temperature. The mixture was stirred at 40 °C under nitrogen atmosphere for 18 h. The progress of the reaction was monitored by LC-MS. Upon completion, the reaction mixture was precipitated by addition of acetone (15 mL). The precipitate was filtered through sintered funnel and washed with diethyl ether, and acetone. The solid was dried under vacuum to obtain compound **13** in 70% yield. ^{1}H NMR (600 MHz, DMSO-*d*$_6$): δ = 12.18 (s, 1H), 12.15 (s, 1H), 10.58 (s, 1H), 10.32 (s, 2H), 9.92 (s, 2H), 9.12 (d, *J* = 8.7 Hz, 1H), 8.96 (d, *J* = 8.4 Hz, 1H), 8.80 (d, *J* = 8.5 Hz, 1H), 8.73 (d, *J* = 8.2 Hz, 1H), 8.63 (s, 1H), 8.55 (d, *J* = 10.8 Hz, 5H), 8.27 (d, *J* = 7.5 Hz,

2H), 8.10 (d, J = 6.8 Hz, 2H), 6.28 (s, 2H), 4.75 (s, 6H), 4.25 (d, J = 5.7 Hz, 2H), 3.31 – 3.29 (m, 2H), 2.62 – 2.58 (m, 2H), 2.52 (s, 6H), 2.43 (s, 6H); ^{13}C NMR (150 MHz, DMSO): δ = 171.45, 169.81, 164.05, 163.88, 153.95 (2C), 148.80, 148.15, 146.24, 146.15, 146.04 (2C), 144.76, 142.46, 141.52 (2C), 139.24, 136.03, 135.97, 135.39 (2C), 135.19, 134.42, 134.21, 133.20, 133.12 (2C), 131.71, 131.13 (2C), 130.76 (2C), 130.44 (2C), 129.60 (2C), 122.86, 122.26 (3C), 122.03, 119.69 (2C), 46.55 (2C), 43.41, 36.52, 24.36, 16.55 (3C), 14.61; MS (ES mass): m/z 462.2 (M+1).

Procedure for preparation methyl 6-((2,9-bis(quinolin-3-ylcarbamoyl)-1,10-phenanthrolin-5-yl)amino)-6-oxohexanoate (15)

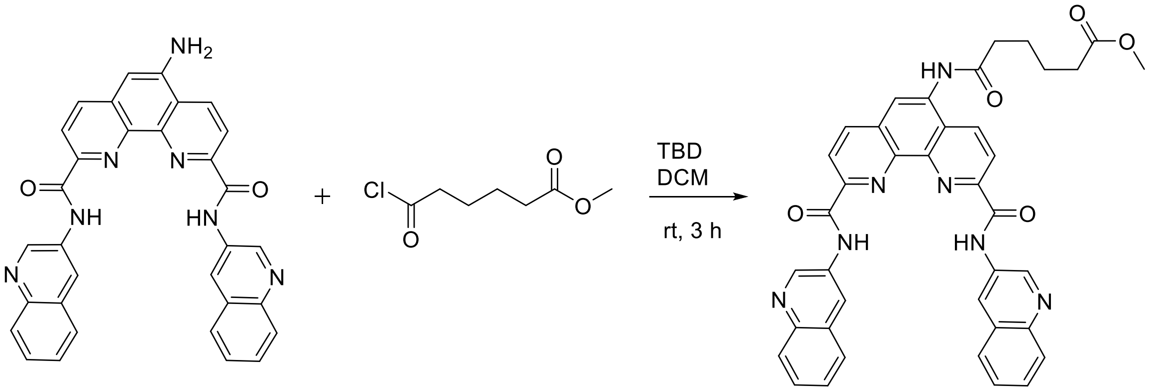

To a stirred solution of 5-amino-N^2,N^9-di(quinolin-3-yl)-1,10-phenanthroline-2,9-dicarboxamide **7** (0.5 g, 0.93 mmol) in dry DCM (15 mL) was added methyl 6-chloro-6-oxohexanoate (0.33 g, 1.86 mmol) and 1,5,7-triazabicyclo[4.4.0]dec-5-ene (0.19 g, 1.4 mmol) at room temperature. The mixture was stirred at room temperature under nitrogen atmosphere for 3 h. The progress of the reaction was monitored by TLC. Upon completion, the reaction mixture was filtered through a sintered funnel. The solid was washed with DCM and diethyl ether then dried under vacuum to give methyl 6-((2,9-bis(quinolin-3-ylcarbamoyl)-1,10-phenanthrolin-5-yl)amino)-6-oxohexanoate **15** in 82% yield as light yellow solid.

^{1}H NMR (600 MHz, DMSO-d_6): δ = 11.87 (s, 1H), 11.83 (s, 1H), 10.38 (s, 1H), 9.70 (d, J = 2.5 Hz, 1H), 9.68 (d, J = 2.5 Hz, 1H), 9.15 (t, J = 2.8 Hz, 2H), 9.04 (d, J = 8.6 Hz, 1H), 8.84 (d, J = 8.3 Hz, 1H), 8.72 (d, J = 8.5 Hz, 1H), 8.63 (d, J = 8.2 Hz, 1H), 8.53 (s, 1H), 8.12 – 8.09 (m, 4H), 7.77 – 7.74 (m, 2H), 7.71 – 7.64 (m, 2H), 3.63 (s, 3H), 2.64 (t, J = 7.2 Hz, 2H), 2.44 (t, J = 7.2 Hz, 2H), 1.79 – 1.69 (m, 4H); ^{13}C NMR (150 MHz, DMSO): δ =

173.76, 172.87, 163.84, 163.65, 149.72, 149.02, 145.94, 145.90, 145.11, 145.05, 144.65, 142.20, 138.64, 134.76, 134.03, 132.91, 132.84, 131.20, 129.16 (2C), 128.81, 128.73, 128.51, 128.49, 128.29, 128.26, 127.64, 127.62, 127.21, 124.23, 124.01, 122.39, 121.65, 121.21, 51.75, 40.53, 33.56, 25.10, 24.61; MS (ES mass): *m*/*z* 678.2 (*M*+1).

Procedure for preparation 6-((2,9-bis(quinolin-3-ylcarbamoyl)-1,10-phenanthrolin-5-yl)amino)-6-oxohexanoic acid (16)

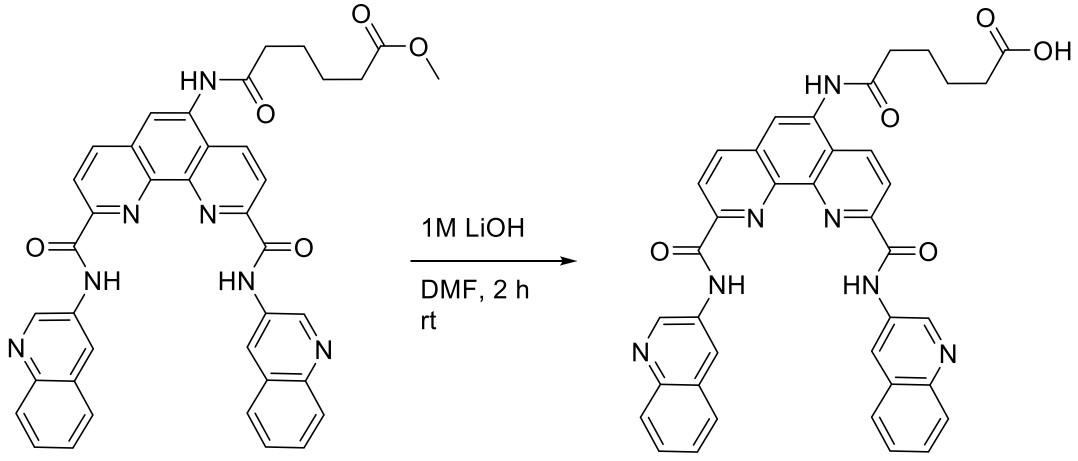

To a stirred solution of methyl 6-((2,9-bis(quinolin-3-ylcarbamoyl)-1,10-phenanthrolin-5-yl)amino)-6-oxohexanoate **15** (0.3 g, 0.44 mmol) in dry DMF (10 mL) was added aqueous 1M LiOH (0.02 g, 0.83 mmol) at room temperature. The mixture was stirred at room temperature under nitrogen atmosphere for 2 h. The progress of the reaction was monitored by TLC. Upon completion, the solvent was removed under reduced pressure then dissolved in 5 mL of water. The mixture was neutralized by slow addition of 1N HCl which resulted in the formation of a solid. The mixture was filtered through sintered funnel and dried under vacuum to give 6-((2,9-bis(quinolin-3-ylcarbamoyl)-1,10-phenanthrolin-5-yl)amino)-6-oxohexanoic acid as yellow solid.

^{1}H NMR (400 MHz, DMSO-d_6): δ = 11.91 (s, 1H), 11.89 (s, 1H), 10.42 (s, 1H), 9.76 (s, 2H), 9.24 (s, 2H), 9.04 (d, *J* = 8.6 Hz, 1H), 8.81 (d, *J* = 8.5 Hz, 1H), 8.69 (d, *J* = 8.4 Hz, 1H), 8.60 (d, *J* = 8.1 Hz, 1H), 8.51 (s, 1H), 8.14 (t, *J* = 9.2 Hz, 5H), 7.81 (t, *J* = 7.7 Hz, 2H), 7.73 (t, *J* = 7.5 Hz, 2H), 2.65 (t, *J* = 7.4 Hz, 2H), 2.35 (t, *J* = 7.2 Hz, 2H), 1.82 – 1.61 (m, 4H); ^{13}C NMR (150 MHz, DMSO): δ = 174.88, 172.97, 163.85, 163.65, 149.48 (2C), 148.78, 144.95, 144.79, 144.58 (2C), 142.15 (2C), 138.62, 134.80, 134.09, 133.06, 132.96,

131.23, 129.63, 128.67 (3C), 128.40, 128.37, 128.13 (2C), 127.75, 127.63, 127.24, 125.90, 122.37, 121.63, 121.21, 36.26, 33.96, 25.22, 24.69; MS (ES mass): *m*/*z* 664.2 (*M*+1).

Procedure for preparation 3,3'-((5-(5-carboxypentanamido)-1,10-phenanthroline-2,9-dicarbonyl)bis(azanediyl))bis(1-methylquinolin-1-ium) diiodide (17)

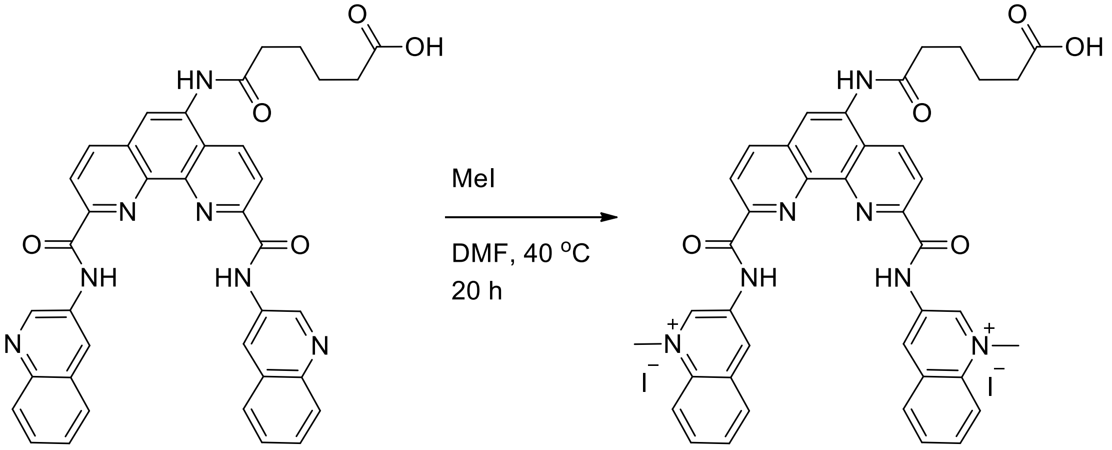

To the solution of 6-((2,9-bis(quinolin-3-ylcarbamoyl)-1,10-phenanthrolin-5-yl)amino)-6-oxohexanoic acid **16** (0.1 g, 0.15 mmol) in a dry DMF (10 mL) was added methyl iodide (0.32 g, 2.26 mmol) at room temperature. The mixture was stirred at 40 °C under nitrogen atmosphere for 20 h. The progress of the reaction was monitored by LC-MS. Upon completion, the reaction mixture was precipitated by addition of acetone (15 mL). The precipitate was filtered through sintered funnel and washed with diethyl ether, and acetone. The resulting solid was dried under vacuum to obtain pure compound **17** as yellow solid in 92% yield.

^{1}H NMR (600 MHz, DMSO-d_6): δ = 12.16 (s, 1H), 12.14 (s, 1H), 12.09 (s, 1H), 10.46 (s, 1H), 10.31 (t, *J* = 2.4 Hz, 2H), 9.91 (d, *J* = 2.6 Hz, 2H), 9.10 (d, *J* = 8.6 Hz, 1H), 8.94 (d, *J* = 8.3 Hz, 1H), 8.81 (d, *J* = 8.5 Hz, 1H), 8.72 (d, *J* = 8.2 Hz, 1H), 8.60 – 8.49 (m, 5H), 8.37 – 8.20 (m, 2H), 8.12 – 8.06 (m, 2H), 4.75 (d, *J* = 4.5 Hz, 6H), 2.65 (t, *J* = 7.3 Hz, 2H), 2.35 (t, *J* = 7.3 Hz, 2H), 1.77 (q, *J* = 7.5 Hz, 2H), 1.69 (q, *J* = 7.5 Hz, 2H); ^{13}C NMR (150 MHz, DMSO): δ = 174.88, 173.04, 164.07, 163.89, 148.70, 148.00, 146.24, 146.14, 144.75, 142.33, 139.15, 136.03, 135.97, 135.38 (2C), 135.17, 134.48, 134.42, 134.35, 133.21, 133.12, 131.79, 130.77, 130.75, 130.46, 130.44, 129.62, 129.60, 127.89, 122.82, 122.09, 121.84, 119.72, 119.69, 46.57 (2C), 36.25, 33.94, 25.20, 24.67; MS (ES mass): *m*/*z* 692.2 (*M*+1).

Synthesis of Peptide (PP) (21)

Solid phase synthesis of the peptide was carried out on SyroI peptide synthesiser, starting on 4 µmol Rink Amide Fmoc-D-Arg (Pbf) tenta gel resin using *N*Fmoc-D-Arg (Pbf), *N*Fmoc-Cha-OH amino acids, HBTU and HOBT as the coupling reagent, DIPEA/NMP and piperidine as bases (Merck Chemicals). Following chain assembly of the Fmoc protected amino acids, the final Fmoc was removed from the peptidylresin. Next, the peptidyl-resin was added to 10 ml of trifluoroacetic acid (TFA), triisopropyl silane (TIPS) and H_2O (15:1:1). After 3 h, the resin was removed by filtration and the peptide was precipitated with ice cold diethyl ether. The peptide was isolated by centrifugation, then dissolved in H_2O and freeze-dried overnight. MS (ES mass): *m*/*z* 551.6 (*M*+1); HPLC integration analysis: 96% pure.

Synthesis of Phen-DC3 linker with PP (22)

Solid phase synthesis of the Phen-DC3 linker with PP was carried out on SyroI peptide synthesiser, starting on 50 µmol Rink Amide Fmoc-D-Arg (Pbf) tenta gel resin using *N*-Fmoc amino acids, HBTU and HOBT as the coupling reagent, DIPEA/NMP and piperidine as bases (Merck Chemicals). Following chain assembly of the Fmoc protected amino acids, the final Fmoc was removed from the peptidylresin with piperidine. Addition of the 3,3'-((5-(5-carboxypentanamido)-1,10-phenanthroline-2,9-dicarbonyl)bis-(azanediyl))bis(1-methylquinolin-1-ium) diiodide to the peptidyl resin was as follows: To the Phen-DC3 linker acid (52 µmol, 50 mg) in a minimum volume of DMF (approx. 400 µl) was added HATU (54 µmol, 21 mg) and DIPEA (79 µmol, 14 µl) at room temperature and the reaction mixture was stirred for 10 min. This solution was added to 42 µmol peptidyl-resin and reacted for 12 h. Next, the Phen-DC3 linker acid peptidyl-resin was added to 2 mL of trifluoroacetic acid (TFA), triisopropyl silane and H_2O (15:1:1). After 3 h, the resin was removed by filtration and this process was repeated 2 times. The peptide was precipitated with ice cold diethyl ether and was isolated by centrifugation and this process was repeated 3 times. The peptide was then dissolved in H_2O, acetonitrile mixture and freeze-dried overnight. MS (ES mass): *m*/*z* 592.7 (*M*+1); HPLC integration analysis: 97% pure.

Procedure for preparation of 3,3'-((5-(6-methoxy-6-oxohexanamido)-1,10-phenanthroline-2,9-dicarbonyl)bis(azanediyl))bis(1-methylquinolin-1-ium) di-trifluoromethanesulfonate (23)

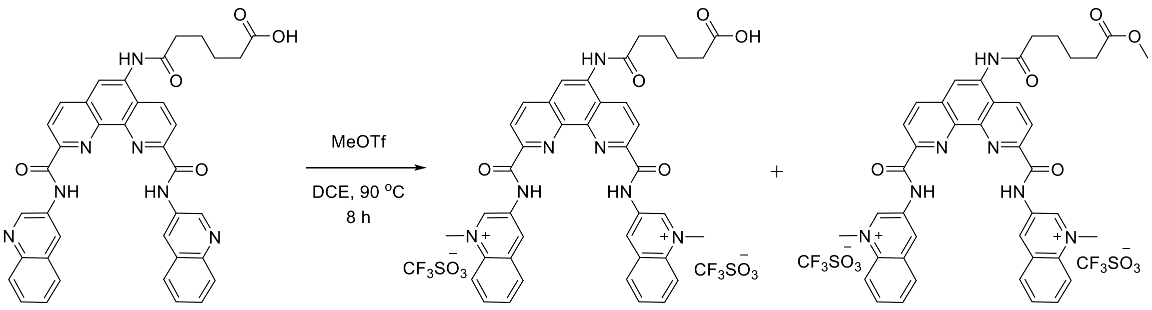

To the solution of 6-((2,9-bis(quinolin-3-ylcarbamoyl)-1,10-phenanthrolin-5-yl)amino)-6-oxohexanoic acid **16** (0.1 g, 0.15 mmol) in a dry DCE (15 mL) was added methyl triflate (0.07 g, 0.45 mmol) at room temperature. The mixture was stirred at 90 °C under nitrogen atmosphere for 8 h. The progress of the reaction was monitored by LC-MS. Upon completion, the reaction mixture was filtered through a sintered funnel and washed with diethyl ether. The obtained solid was purified using preparative reverse phase HPLC to give **23** in 27% yield.

^{1}H NMR (600 MHz, DMSO-d_6): δ = 12.19 (s, 1H), 12.16 (s, 1H), 10.49 (s, 1H), 10.32 (d, *J* = 2.6 Hz, 2H), 9.91 (d, *J* = 2.7 Hz, 2H), 9.10 (d, *J* = 8.6 Hz, 1H), 8.93 (d, *J* = 8.3 Hz, 1H), 8.80 (d, *J* = 8.5 Hz, 1H), 8.72 (d, *J* = 8.2 Hz, 1H), 8.58 – 8.53 (m, 5H), 8.28 – 8.25 (m, 2H), 8.11 – 8.08 (m, 2H), 4.75 (s, 6H), 3.63 (s, 3H), 2.65 (t, *J* = 4.0 Hz, 2H), 2.44 (t, *J* = 4.0 Hz, 2H), 1.79 – 1.68 (m, 4H); ^{13}C NMR (150 MHz, DMSO): δ = 173.76, 172.98, 164.09, 163.91, 158.05, 148.71, 148.01, 146.25, 146.15, 144.75, 142.33, 139.11, 136.02, 135.96, 135.39, 135.32, 135.17, 134.47, 134.41, 134.33, 133.24, 133.14, 131.77, 130.76, 130.73, 130.45, 130.43, 129.62, 129.60, 127.85, 122.80, 122.05, 121.78, 119.69, 119.67, 70.25 (2C), 51.75, 33.56, 25.09, 24.60; MS (ES mass): *m*/*z* 353.8 (*M*+1); HPLC integration analysis: 94% pure.

References

1. Jamroskovic, J.; Doimo, M.; Chand, K.; Obi, I.; Kumar, R.; Brännström, K.; Hedenström, M.; Das, R. N.; Akhunzianov, A.; Deiana, M.; Kasho, K.; Sato, S. S.; Pourbozorgi, P. L.; Mason, J. E.; Medini, P.; Öhlund, D.; Wanrooij, S.; Chorell, E.; Sabouri, N. Quinazoline Ligands Induce Cancer Cell Death through Selective STAT3 Inhibition and G-Quadruplex Stabilization. *J. Am. Chem. Soc.* 2020, 142, 6, 2876-2888
2. Li Z, Liu B, Jin W, Wu X, Zhou M, Liu VZ, Goel A, Shen Z, Zheng L, Shen B. hDNA2 nuclease/helicase promotes centromeric DNA replication and genome stability. *EMBO J.* 2018 Jul 13;37(14):e96729.
3. R. C. Blake, A. R. Pavlov, M. Khosraviani, H. E. Ensley, G. E. Kiefer, H. Yu, X. Li and D. A. Blake, *Bioconjugate Chem.*, 2004, **15**, 1125-1136.
4. E. Chorell, J. S. Pinkner, C. Bengtsson, S. Edvinsson, C. K. Cusumano, E. Rosenbaum, L. B. A. Johansson, S. J. Hultgren and F. Almqvist, *Chem. Eur. J.*, 2012, **18**, 4522-4532.

NMR Spectra

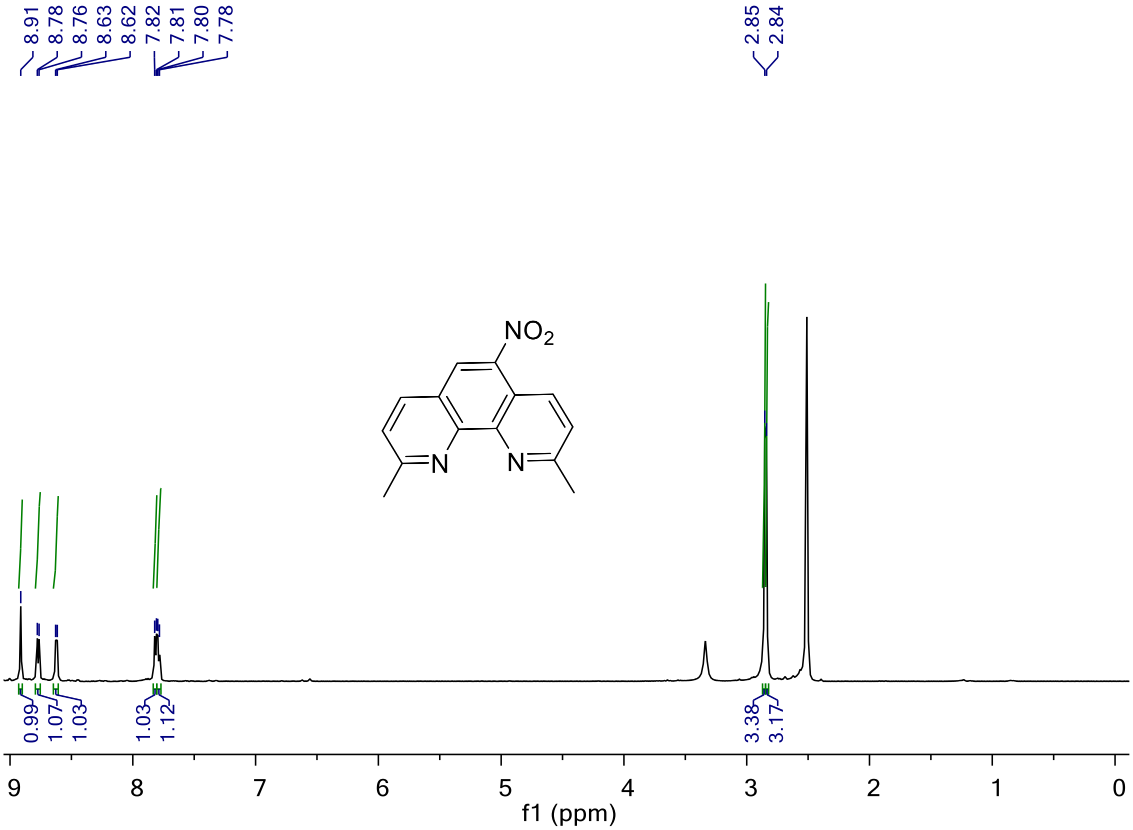

^{1}H NMR of **2** (d^6-DMSO, 600 MHz)

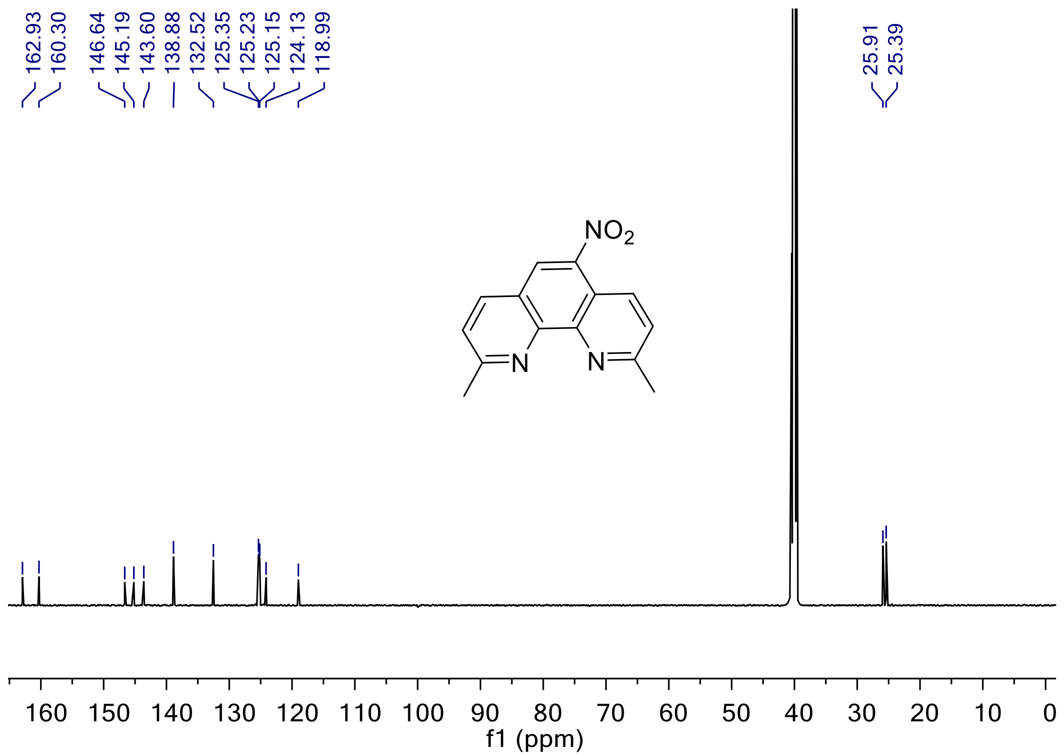

^{13}C NMR of **2** (d^6-DMSO, 150 MHZ)

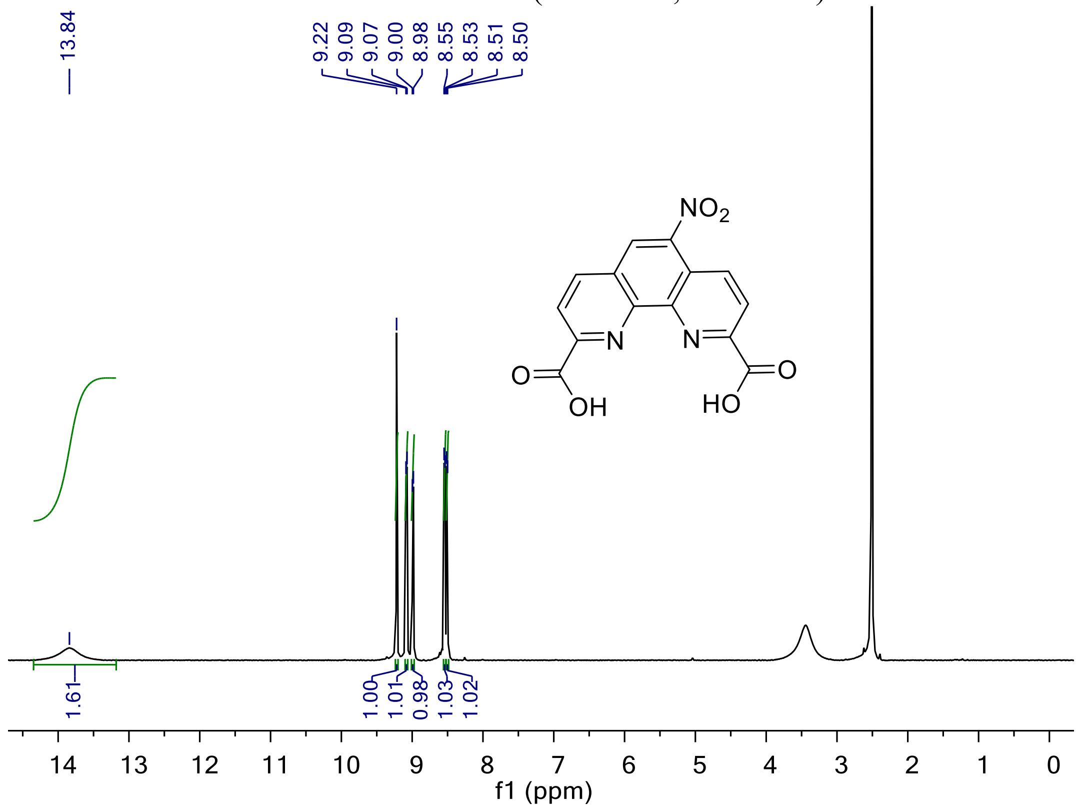

^{1}H NMR of **4** (d^6-DMSO, 600 MHz)

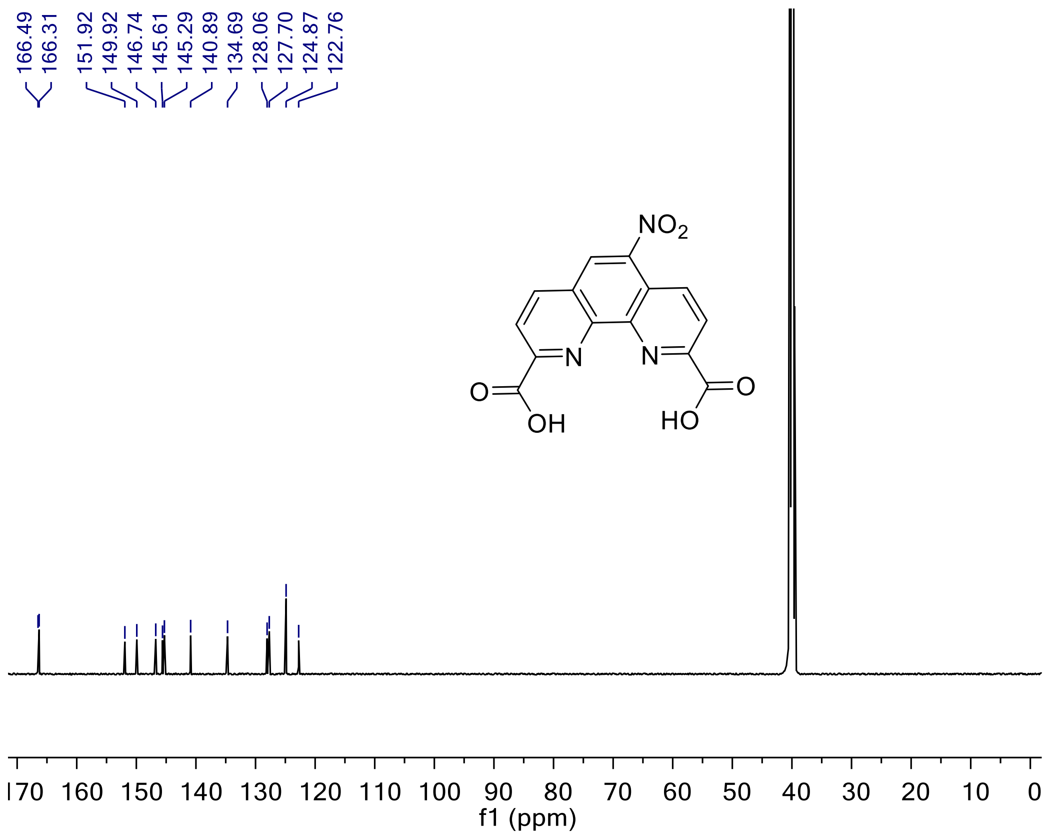

^{13}C NMR of **4** (d^6-DMSO, 150 MHZ)

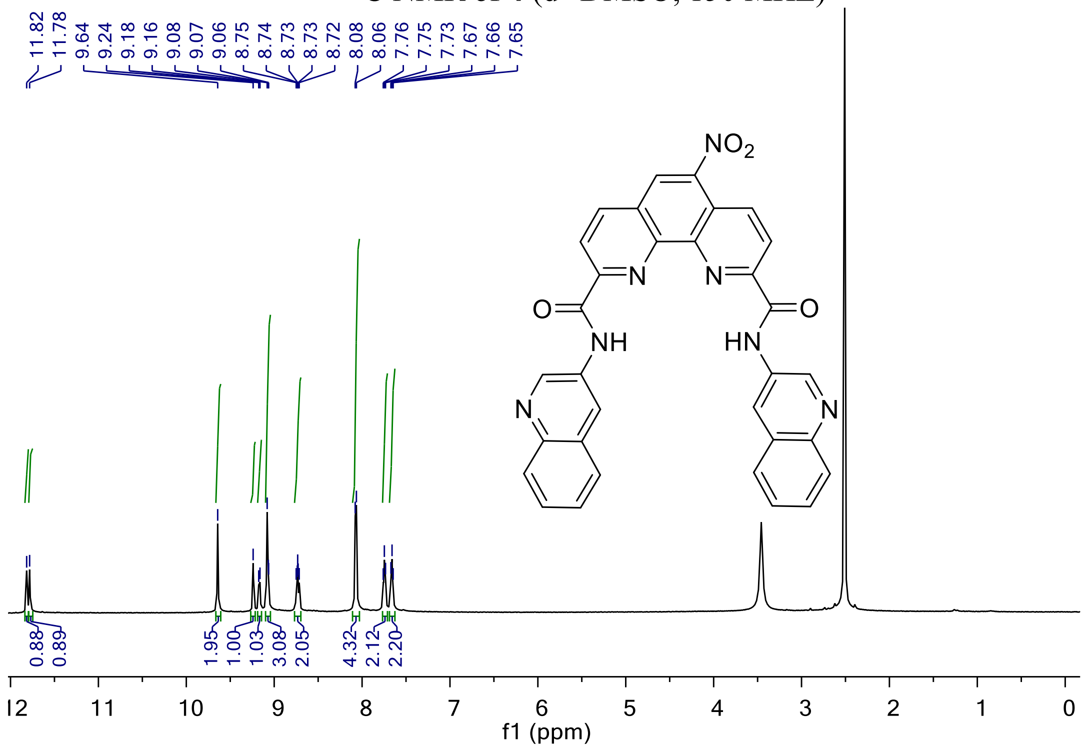

^{1}H NMR of **5** (d^6-DMSO, 600 MHz)

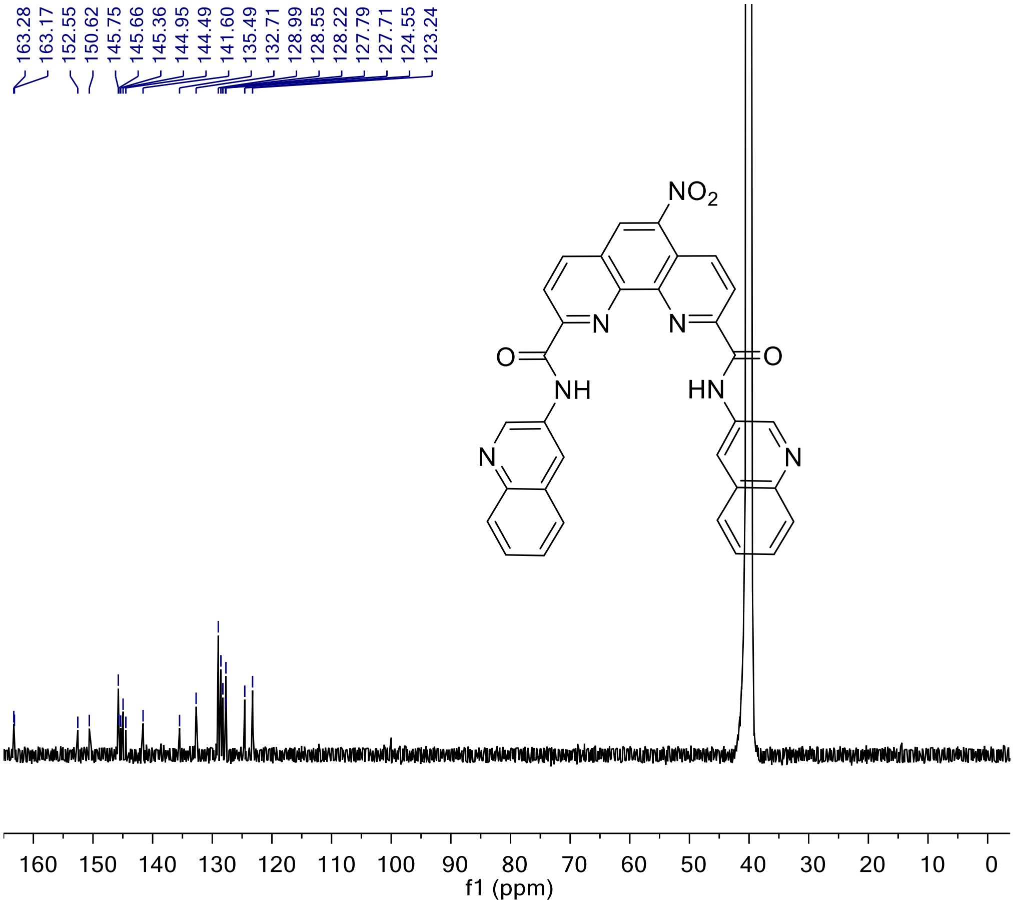

^{13}C NMR of **5** (d^6-DMSO, 150 MHZ)

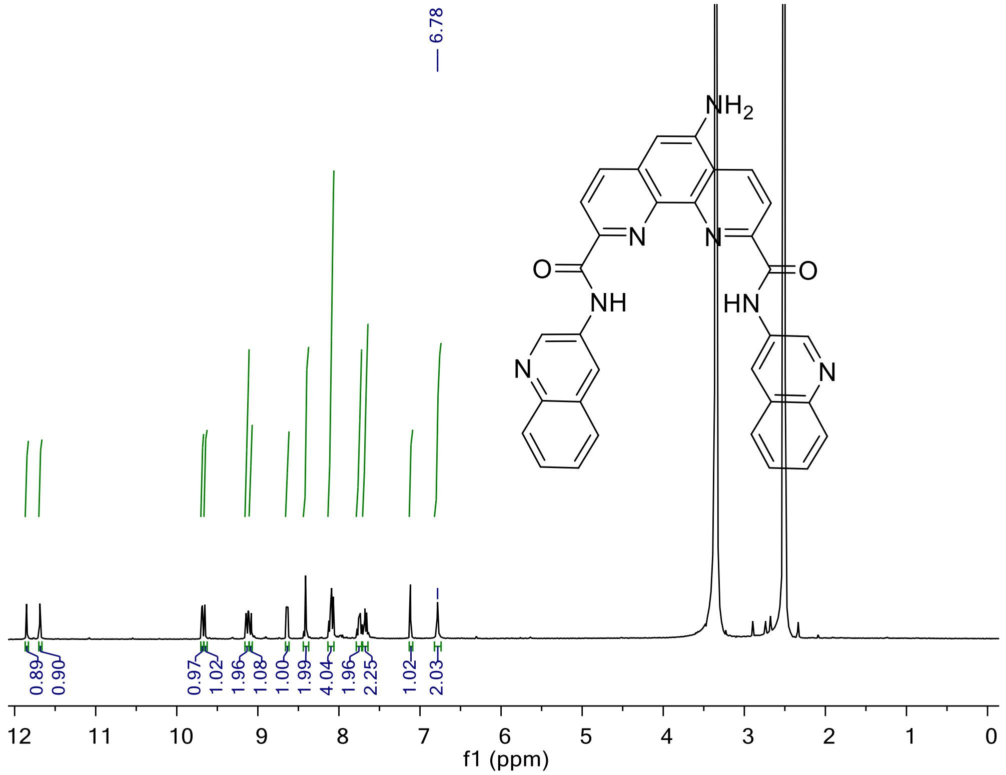

^{1}H NMR of **6** (d[6]-DMSO, 400 MHz)

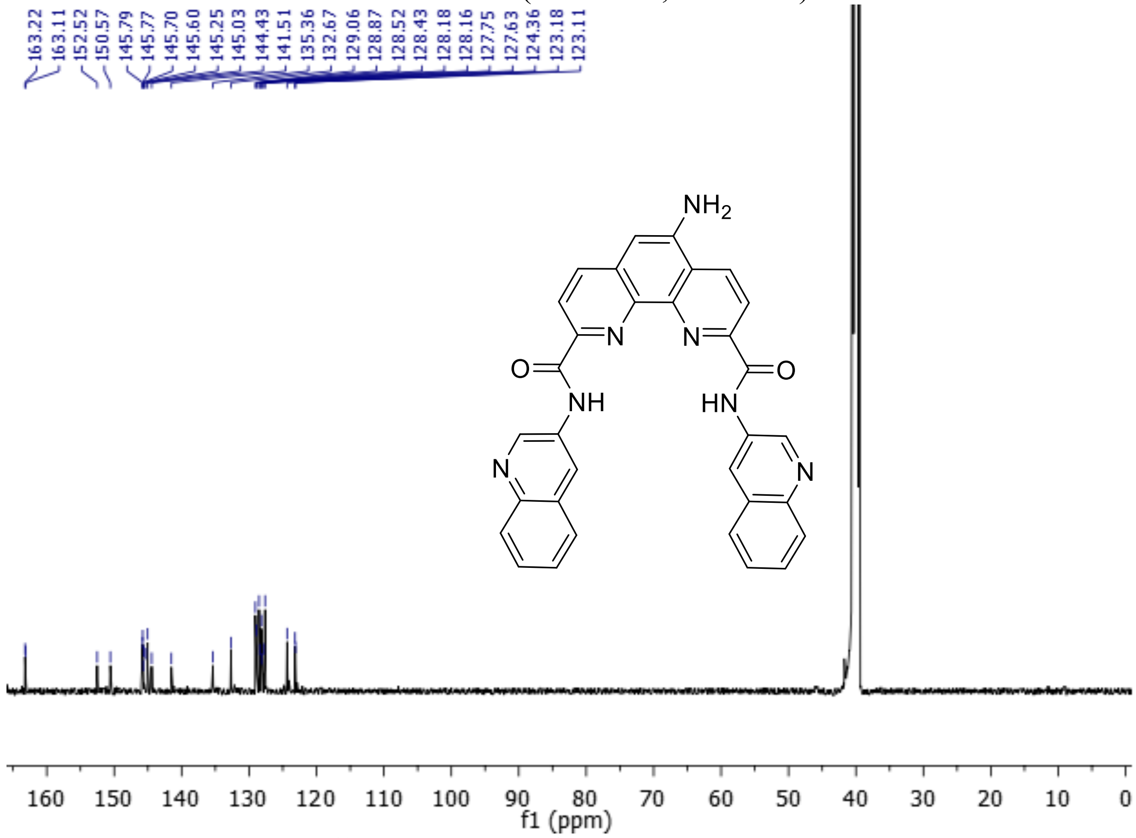

^{13}C NMR of **6** (d[6]-DMSO, 150 MHZ)

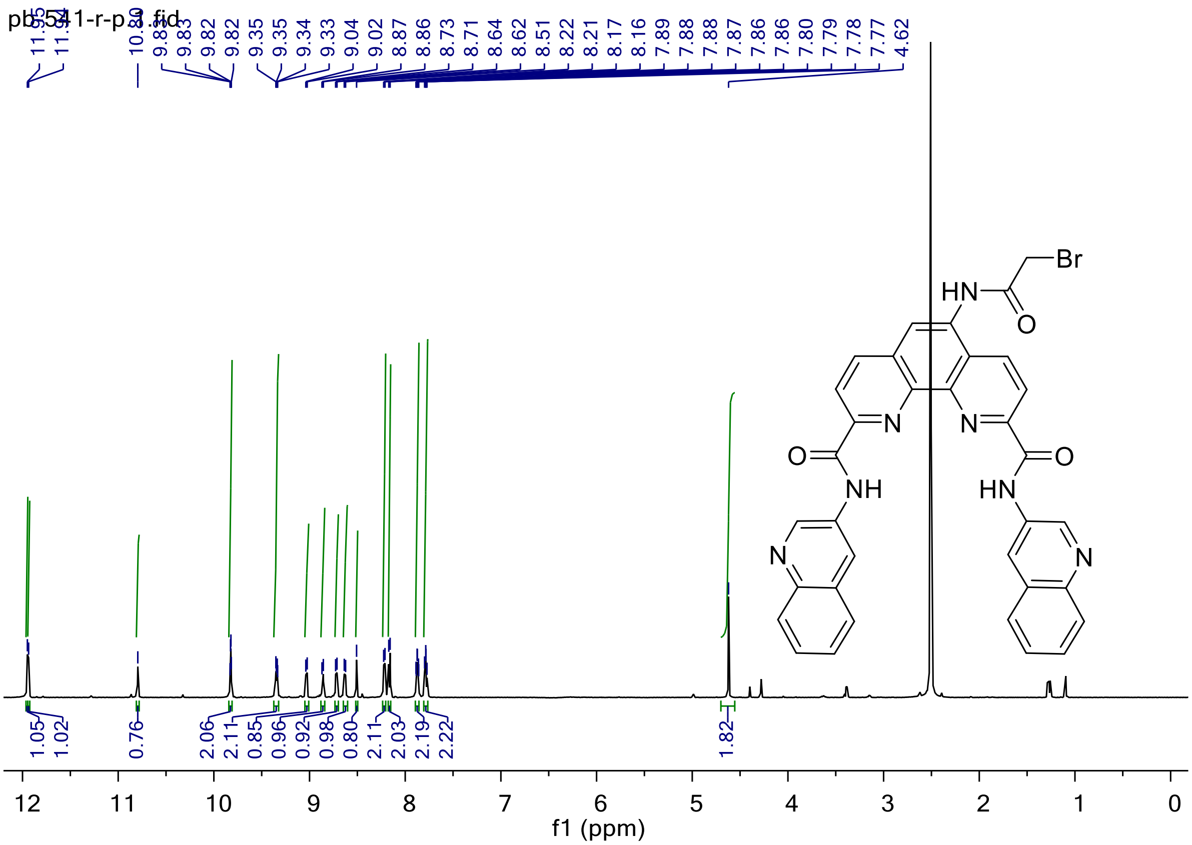

^{1}H NMR of **8** (d^6-DMSO, 600 MHz)

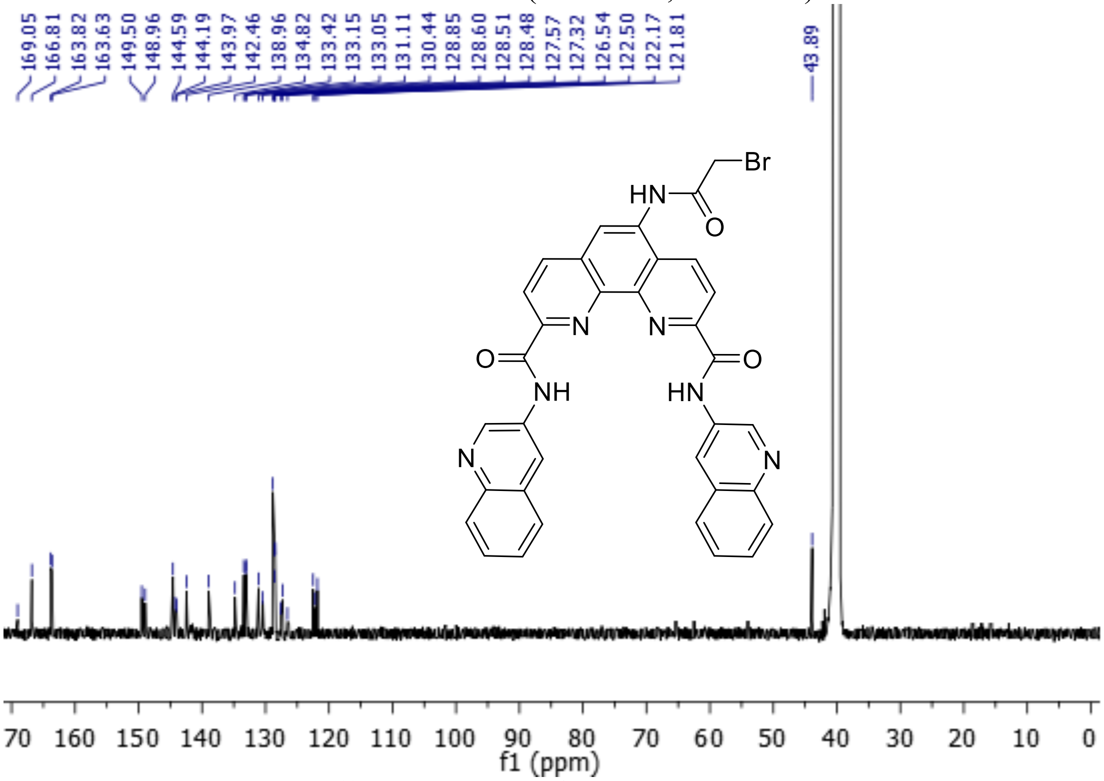

^{13}C NMR of **8** (d^6-DMSO, 150 MHZ)

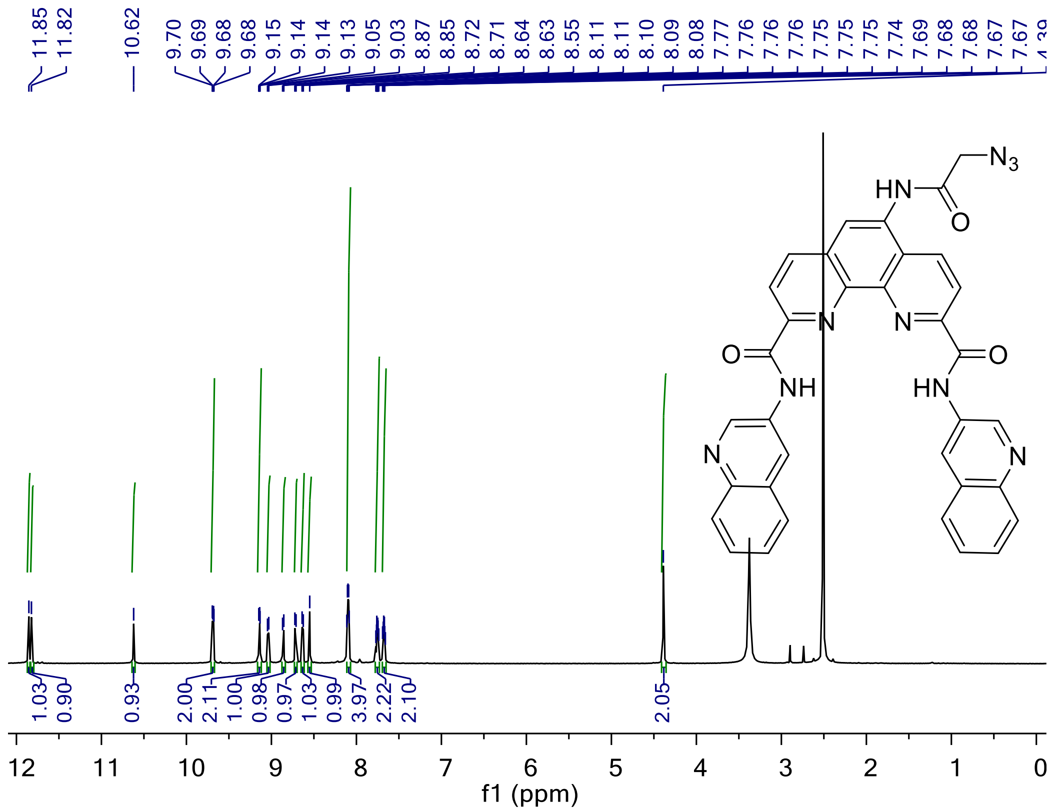

^{1}H NMR of **9** (d^{6}-DMSO, 600 MHz)

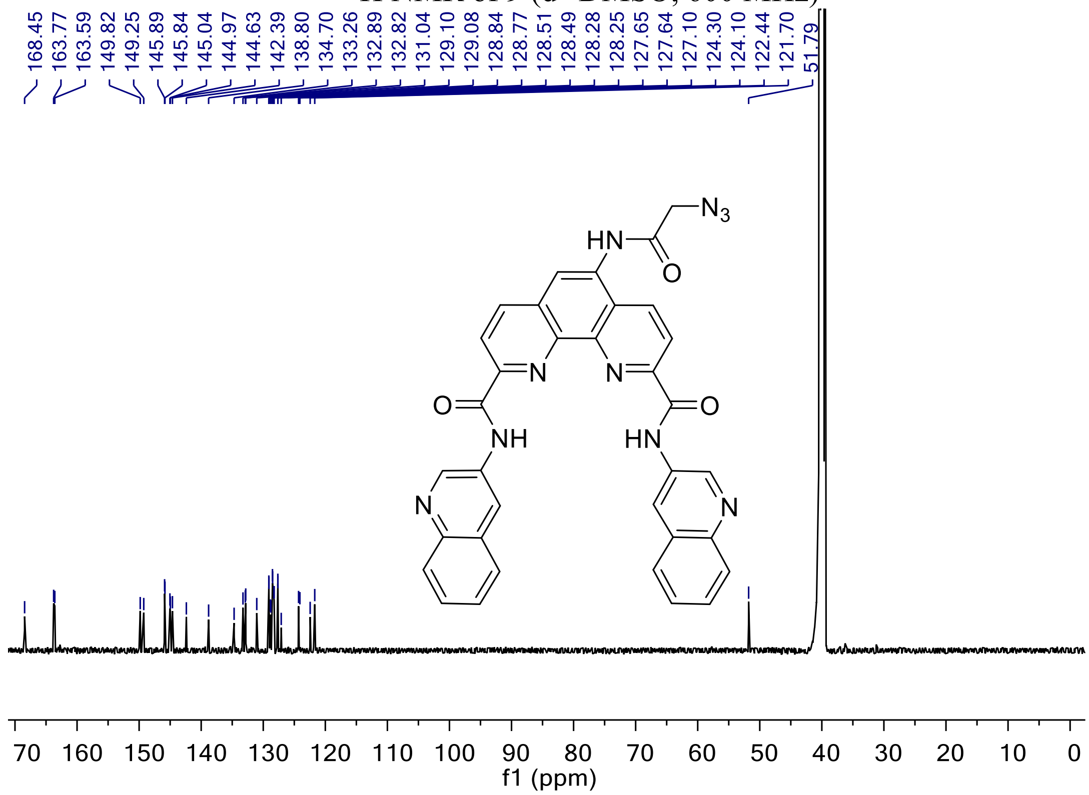

^{13}C NMR of **8** (d^{6}-DMSO, 150 MHZ)

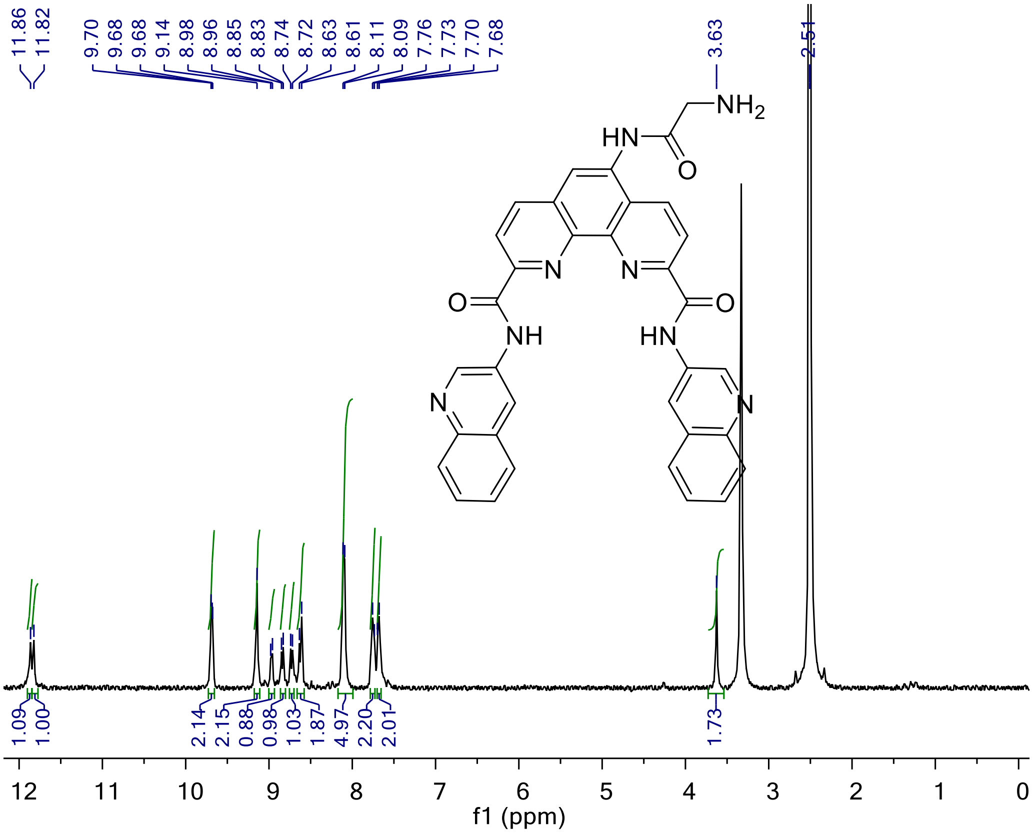

^{1}H NMR of **10** (d^6-DMSO, 400 MHz)

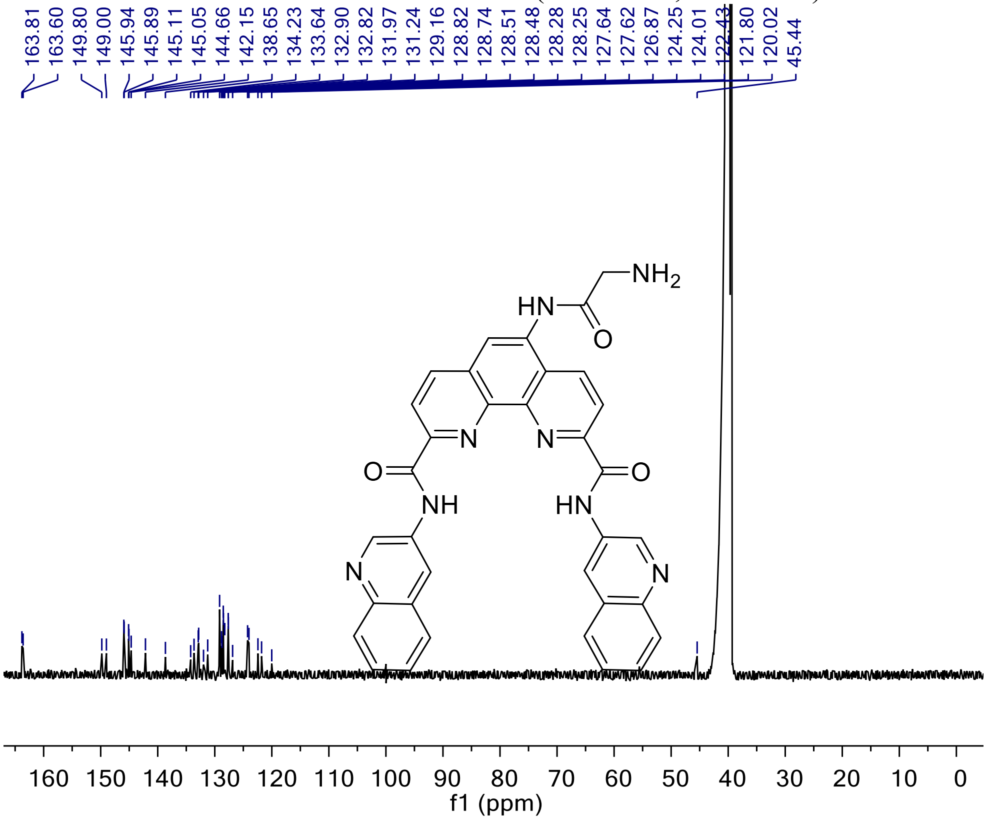

^{13}C NMR of **10** (d^6-DMSO, 150 MHZ)

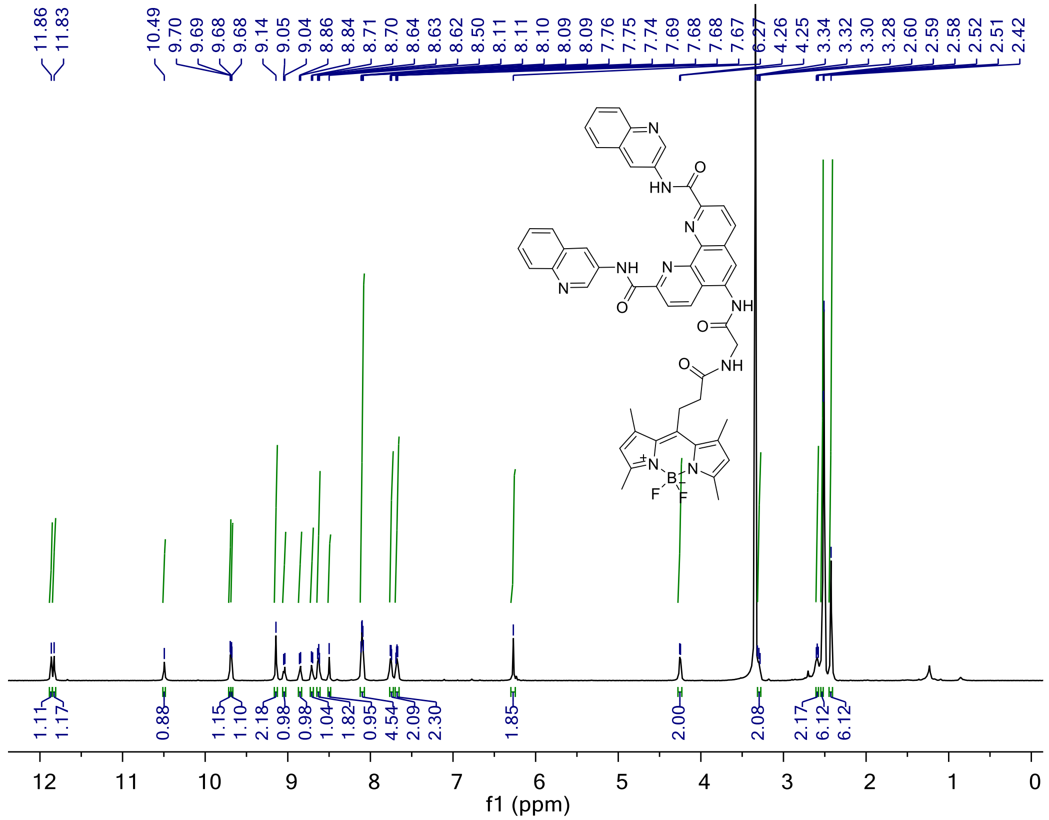

^{1}H NMR of 1**2** (d^{6}-DMSO, 600 MHz)

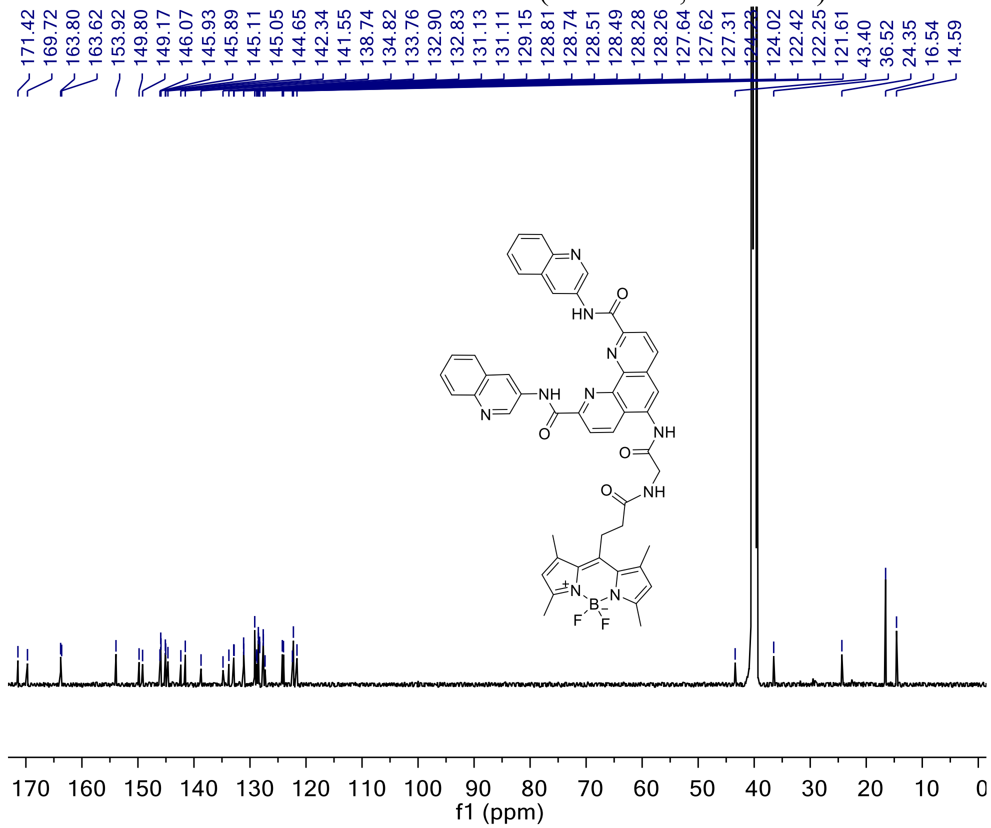

^{13}C NMR of 1**2** (d^{6}-DMSO, 150 MHZ)

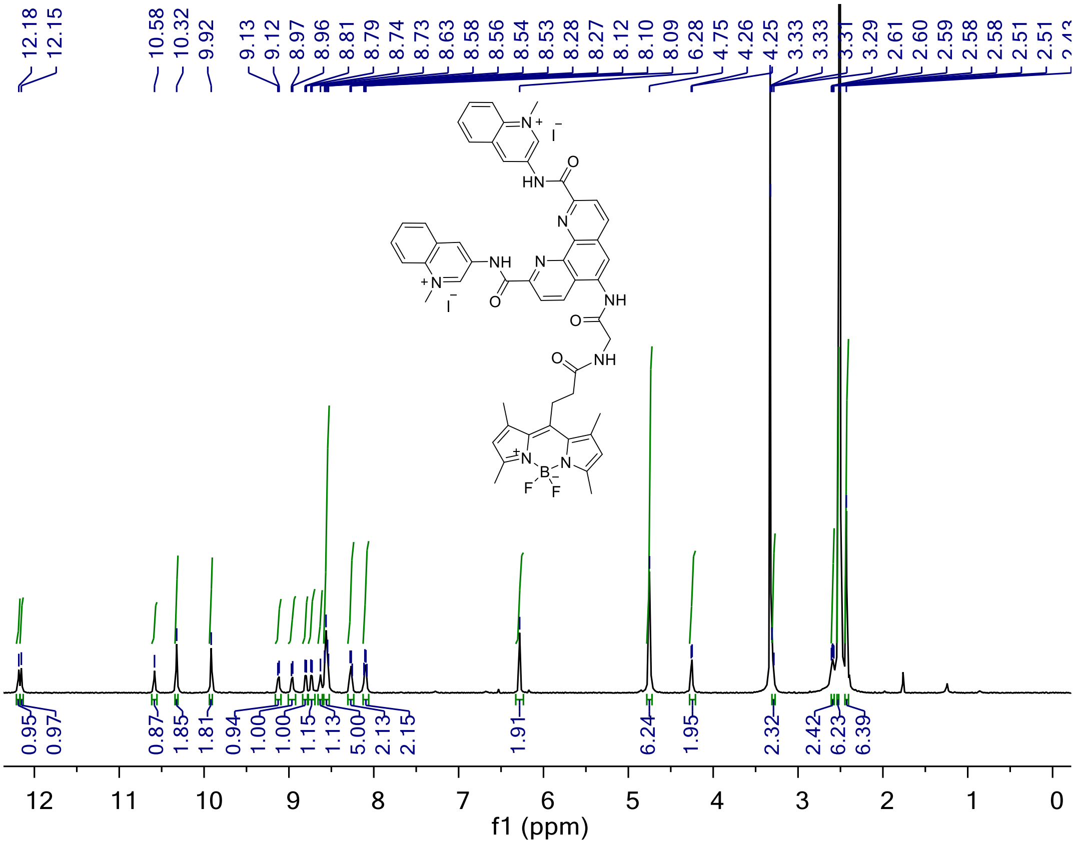

^{1}H NMR of **13** (d[6]-DMSO, 600 MHz)

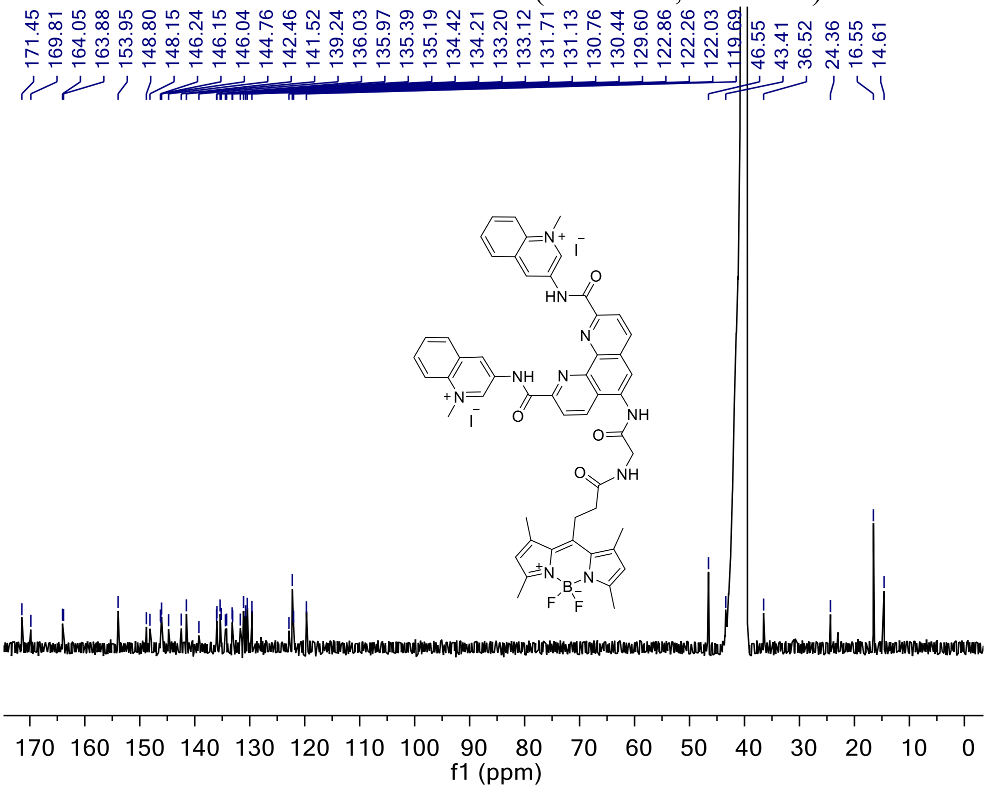

^{13}C NMR of **13** (d[6]-DMSO, 150 MHZ)

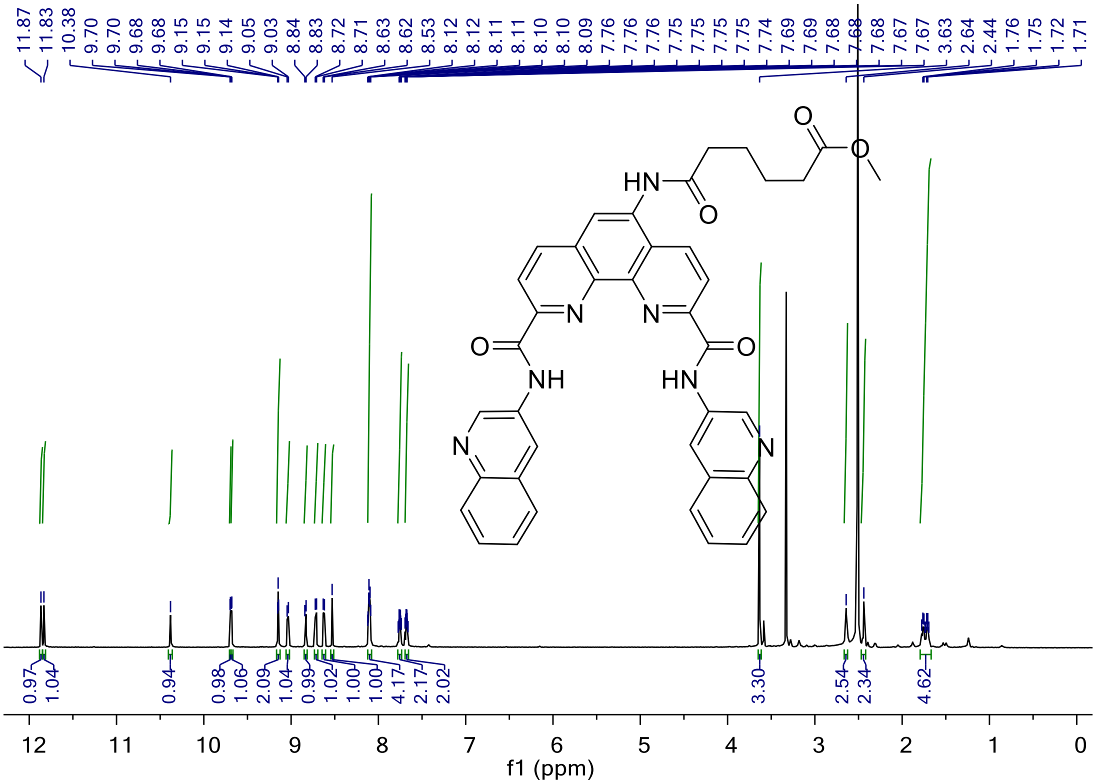

^{1}H NMR of **15** (d^6-DMSO, 600 MHz)

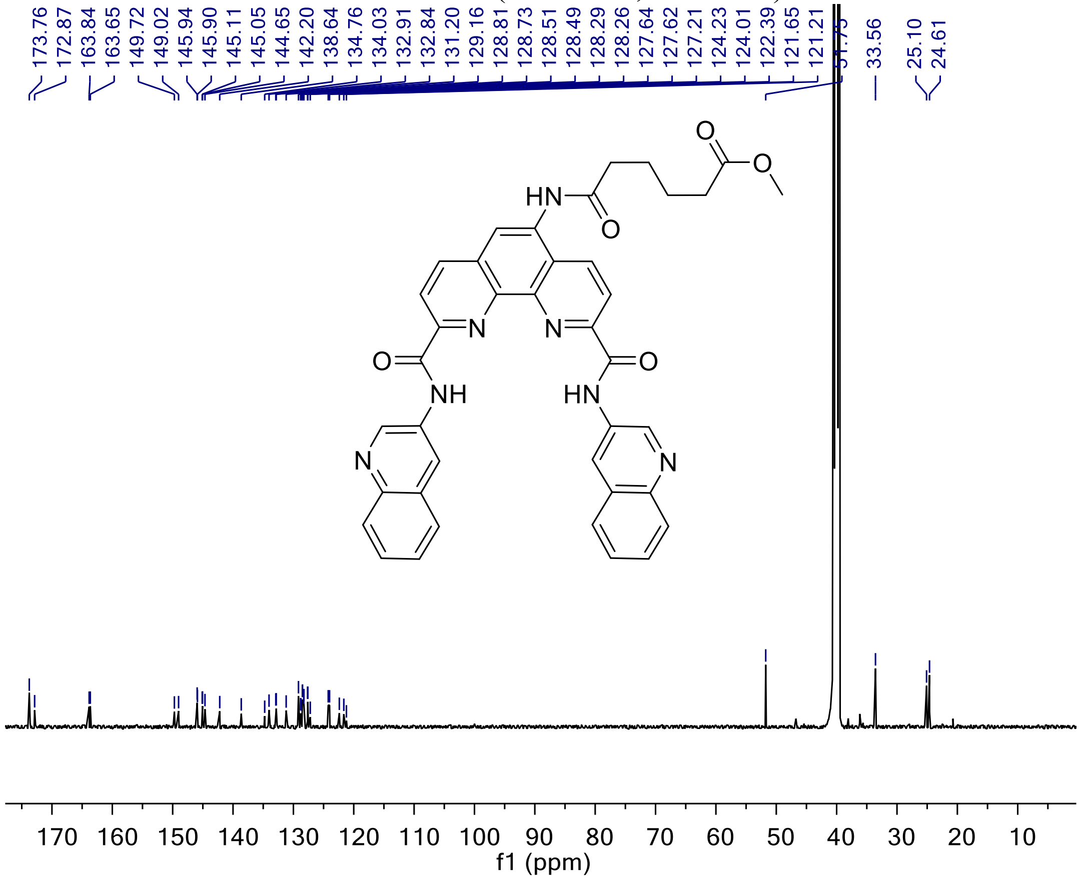

^{13}C NMR of **15** (d^6-DMSO, 150 MHZ)

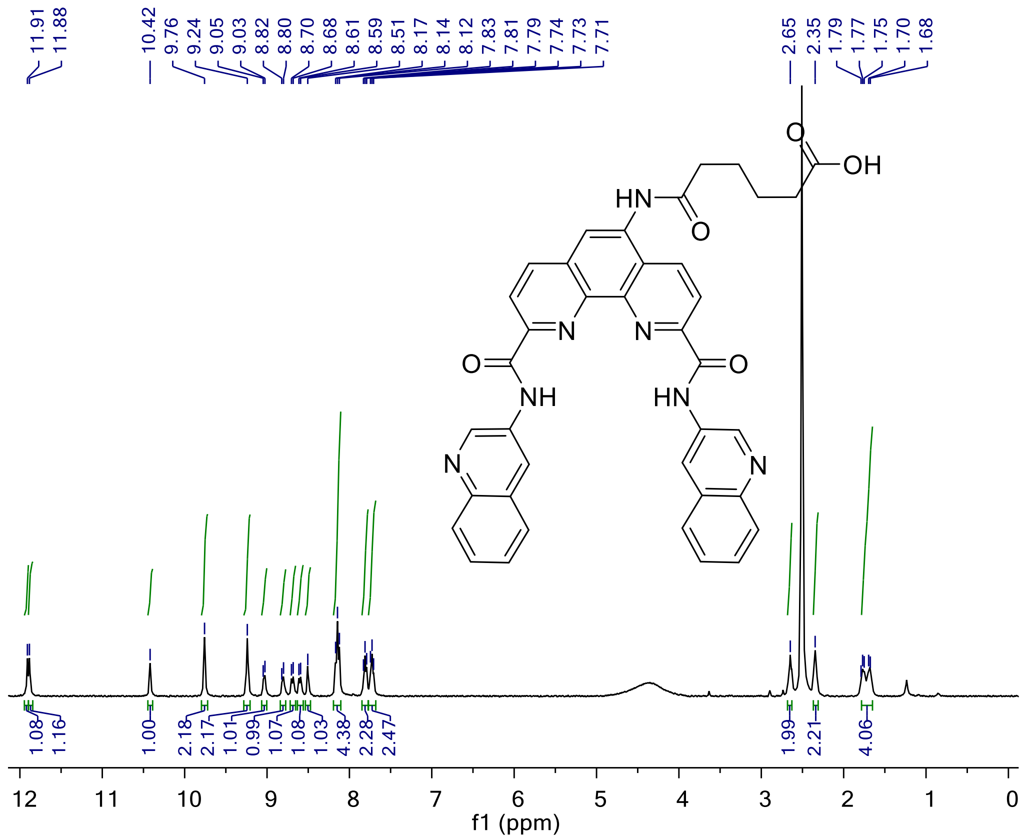

^{1}H NMR of **16** (d^6-DMSO, 600 MHz)

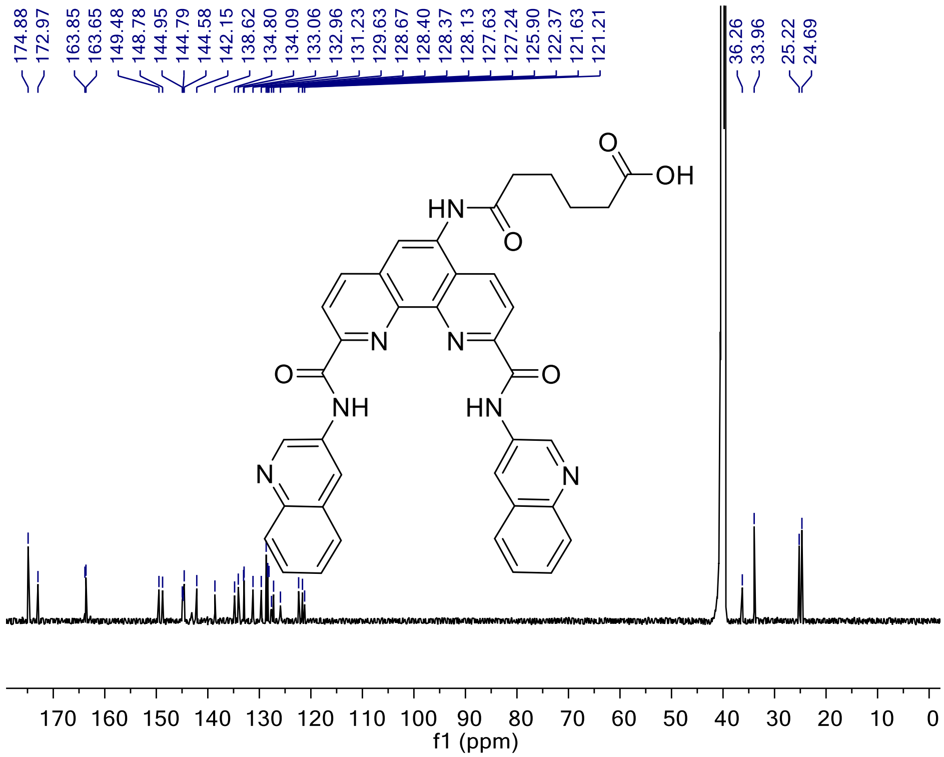

^{13}C NMR of **16** (d^6-DMSO, 150 MHZ)

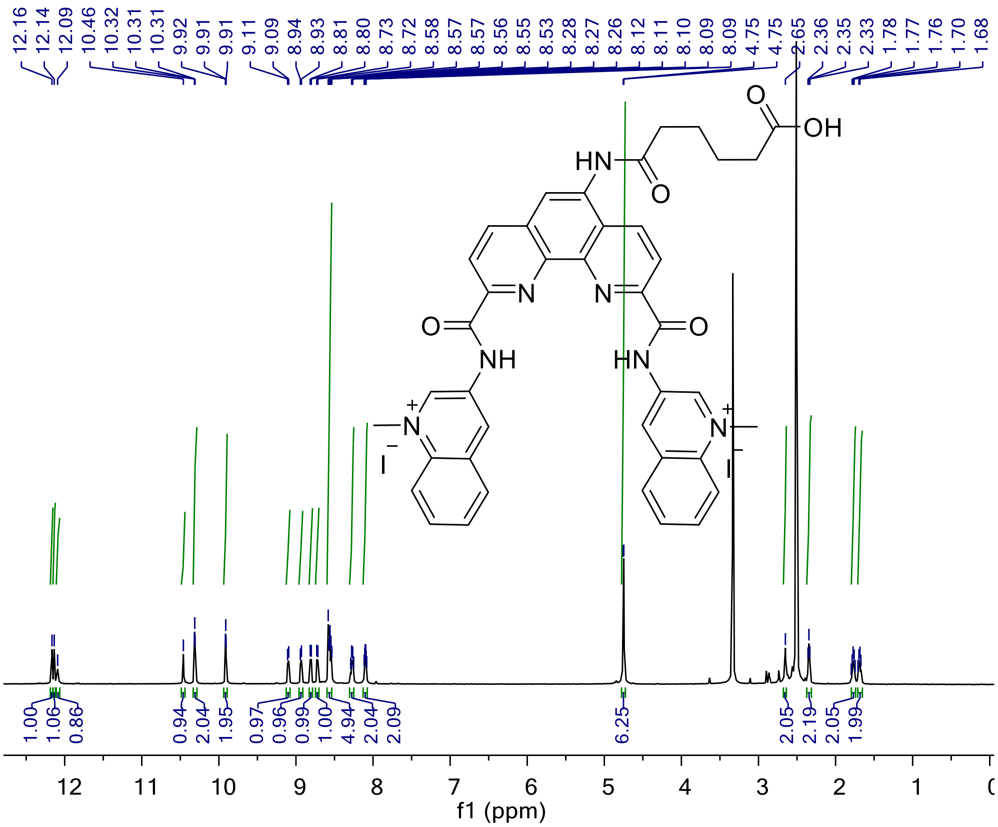

^{1}H NMR of **17** (d^6-DMSO, 600 MHz)

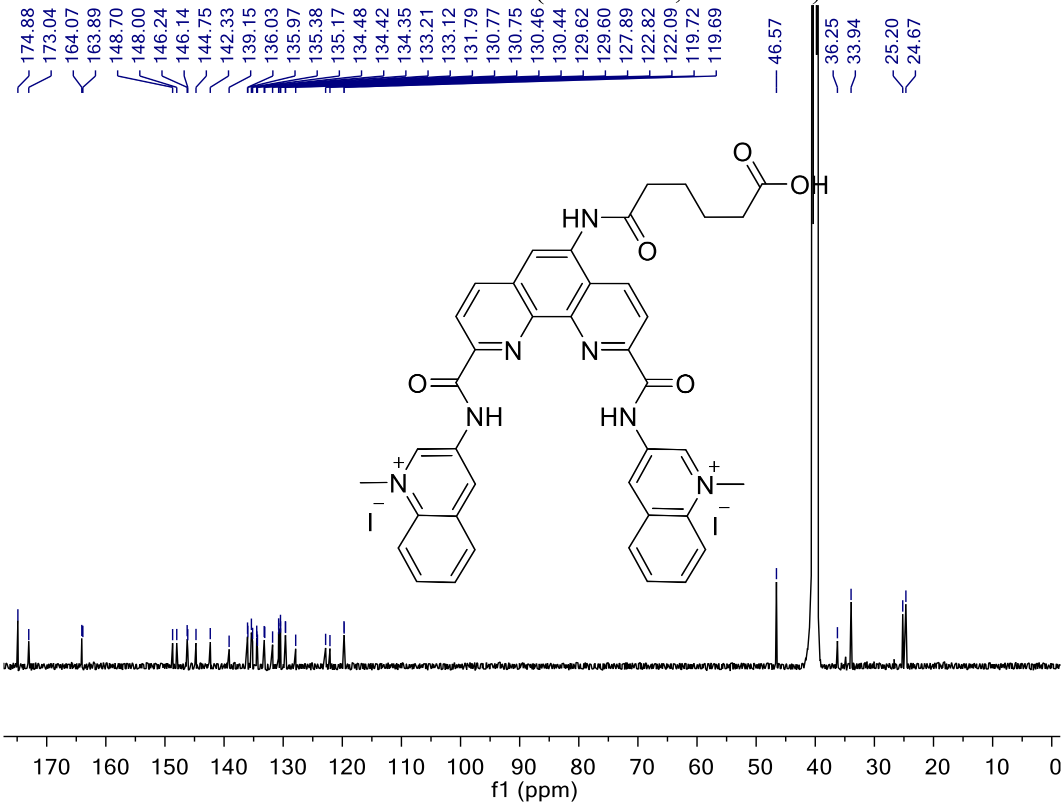

^{13}C NMR of **17** (d^6-DMSO, 150 MHZ)

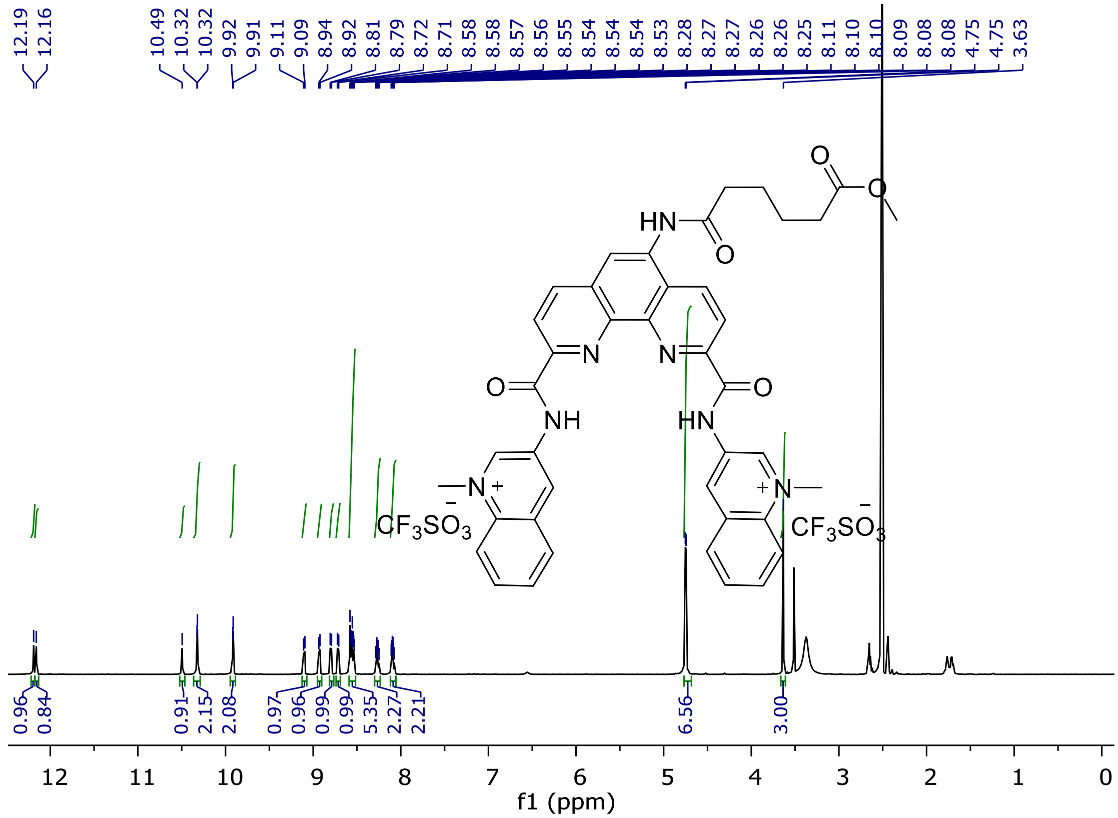

^{1}H NMR of **23** (d^6-DMSO, 600 MHz)

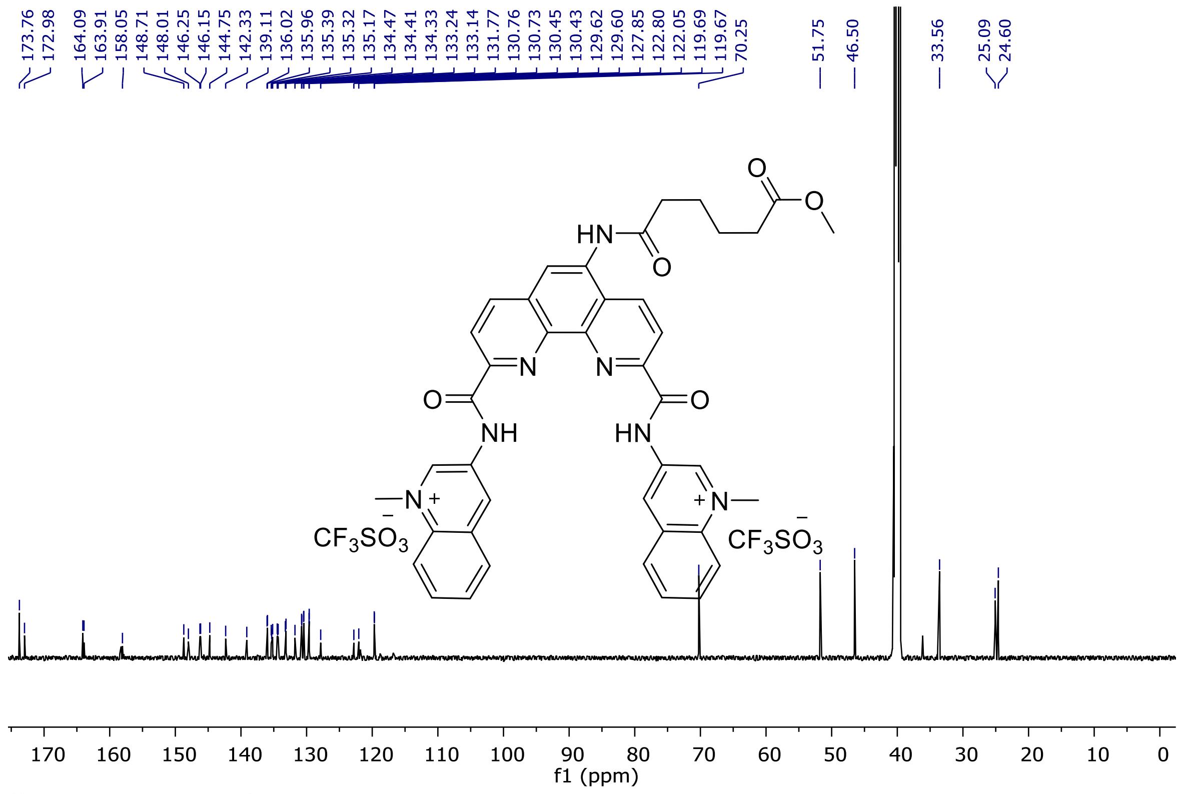

^{13}C NMR of **23** (d^6-DMSO, 150 MHZ)
